# The transcriptional coregulator GRIP1 controls macrophage polarization and metabolic homeostasis

**DOI:** 10.1038/ncomms12254

**Published:** 2016-07-28

**Authors:** Maddalena Coppo, Yurii Chinenov, Maria A. Sacta, Inez Rogatsky

**Affiliations:** 1 The David Rosensweig Genomics Center, Hospital for Special Surgery, 535 East 70th Street, New York, New York 10021, USA; 2 Weill Cornell/Rockefeller/Sloan-Kettering Tri-Institutional MD-PhD Program, 1300 York Avenue, New York, New York 10021, USA; 3 Graduate Program in Immunology and Microbial Pathogenesis, Weill Cornell Graduate School of Medical Sciences, 1300 York Avenue, New York, New York 10021, USA; 4 Department of Microbiology and Immunology, Weill Medical College of Cornell University, 1300 York Avenue, New York, New York 10021, USA

## Abstract

Diet-induced obesity causes chronic macrophage-driven inflammation in white adipose tissue (WAT) leading to insulin resistance. WAT macrophages, however, differ in their origin, gene expression and activities: unlike infiltrating monocyte-derived inflammatory macrophages, WAT-resident macrophages counteract inflammation and insulin resistance, yet, the mechanisms underlying their transcriptional programming remain poorly understood. We recently reported that a nuclear receptor cofactor—glucocorticoid receptor (GR)-interacting protein (GRIP)1—cooperates with GR to repress inflammatory genes. Here, we show that GRIP1 facilitates macrophage programming in response to IL4 via a GR-independent pathway by serving as a coactivator for Kruppel-like factor (KLF)4—a driver of tissue-resident macrophage differentiation. Moreover, obese mice conditionally lacking GRIP1 in macrophages develop massive macrophage infiltration and inflammation in metabolic tissues, fatty livers, hyperglycaemia and insulin resistance recapitulating metabolic disease. Thus, GRIP1 is a critical regulator of immunometabolism, which engages distinct transcriptional mechanisms to coordinate the balance between macrophage populations and ultimately promote metabolic homeostasis.

## Abstract (web summary)

GRIP1 cooperates with the glucocorticoid receptor to repress inflammatory genes. Here the authors show that GRIP1 also controls macrophage polarization, by promoting KLF4-driven activation in response to IL-4, and that mice lacking GRIP1 in macrophages develop severe metabolic dysfunction on a high-fat diet.

## Graphical abstract

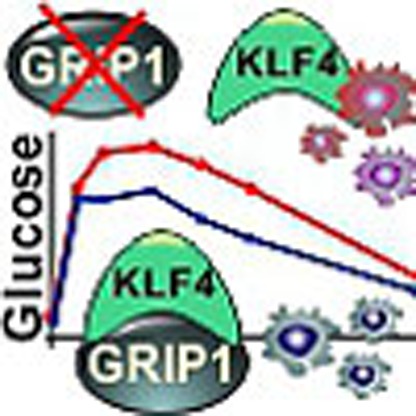

Obesity-associated metabolic syndrome is a cluster of conditions including insulin resistance, hyperinsulinemia, impaired glucose tolerance, dyslipidemia, hypertension and excess body fat that together are associated with increased risk for cardiovascular disease, nonalcoholic fatty liver disease (NAFLD) and type 2 diabetes mellitus (T2DM)^1^,^2^. The causes of obesity-associated metabolic disease reflect a complicated interplay of genetic, environmental and social factors which ultimately disrupt the balance between energy intake and expenditure. Regardless of aetiology, obesity is associated with a state of chronic low-grade inflammation in different tissues, including white adipose tissue (WAT), liver and vascular endothelium^3^,^4^. Indeed, the role of pro-inflammatory cytokines such as tumour necrosis factor (TNF) and interleukin (IL)1β and chemokines (CCL2, CCL5) in disease pathogenesis is well established^5^. Numerous studies have highlighted the central role of AT-associated infiltrating macrophages (ATMΦ) in perpetuation of WAT inflammatory state eventually leading to systemic insulin resistance in mouse models and in humans^6^,^7^.

Notably, ATMΦ comprise diverse populations of cells that differ in their functional characteristics, phenotypic features, intracellular metabolic state and even developmental origin. Despite their inherent plasticity and ability to adjust phenotypes to different environmental cues, ATMΦ can be broadly categorized into monocyte-derived inflammatory, which have been historically referred to as ‘classically activated' or ‘M1' and the tissue-resident macrophages originally termed ‘alternatively activated' or ‘M2' (ref. 3). Tissue-resident macrophages are derived directly from the yolk sac or foetal liver and present in both lean and obese WAT where they support adipose homeostasis, suppress inflammation and promote local insulin sensitivity^3^. Conversely, the monocyte-derived macrophages abundantly infiltrate WAT during obesity where they form ring-like structures around dying adipocytes and secrete inflammatory mediators^8^. As pathology progresses, inflammatory macrophages engulf lipids released by necrotic adipocytes and undergo transformation into multinucleated giant cells that further exacerbate inflammation. These two macrophage populations are phenotypically distinct: although both are characterized by the surface markers cluster of differentiation (CD)11b and F4/80, inflammatory infiltrating macrophages express CD11c, whereas resident macrophages express predominantly CD206 (mannose receptor (MRC)1)^9^,^10^. The key transcriptional networks in the two macrophage populations also differ: infiltrating macrophage transcriptome is dominated by nuclear factor (NF)κB which drives the production of inflammatory mediators^11^, whereas resident macrophages are programmed by coordinated actions of STAT6, Kruppel-like factor (KLF)4 and a nuclear receptor (NR) peroxisome proliferator-activated receptor (PPAR)γ^12^,^13^,^14^,^15^,^16^. Several cytokines and growth factors have been implicated in the programming of the two phenotypes: exposure to interferon γ and lipopolysaccharide (LPS) shifts the balance towards inflammatory macrophages, whereas IL4 or IL13 stimulation leads to resident macrophages-like phenotype^17^,^18^. In addition, IL10 and glucocorticoid hormones (GCs) induce macrophage transcriptomes *in vitro* similar to that programmed by IL4 (ref. 19) consistent with the anti-inflammatory effects of these signals.

GCs in particular have long been known as modulators of macrophage properties: acting through the glucocorticoid receptor (GR), a transcription factor of the NR superfamily, they potently suppress macrophage-mediated inflammation by both activation of anti-inflammatory genes and direct repression of genes encoding inflammatory mediators^20^. After hormone binding, GR translocates into the nucleus and either binds directly to specific palindromic DNA sequences known as GC response elements (GREs) or ‘tethers' to DNA through protein–protein interactions with other DNA-bound factors to ultimately modulate gene transcription. Although in each case GR can either activate or repress transcription depending on the GRE sequence and composition of other DNA-bound regulators, typically, binding to ‘conventional' palindromic GREs leads to activation of associated genes, whereas GR tethering to AP1 or NFκB usually attenuates transcription of their target genes. GR elicits transcriptional changes by recruiting multiple cofactors that act on components of basal machinery or chromatin. From over 100 GR-interacting cofactors, the members of the p160 family— SRC1/Ncoa1, GRIP1/TIF2/SRC2/Ncoa2 (hereafter, GRIP1) and SRC3/Ncoa3—are well established primary coactivators that serve as binding platforms for numerous secondary cofactors, commonly, with chromatin-modifying and remodelling activities^21^,^22^. All three p160s are modular proteins which are recruited by ligand-activated GR to palindromic GREs via their conserved NR interaction domain and facilitate transcriptional activation via their activation domains (AD) 1 and 2.

Interestingly, GRIP1, unlike other p160s, can also function as a GR corepressor at tethering sites relying on a unique repression domain: lack of GRIP1 in macrophages profoundly altered their transcriptome leading to a defective GR-mediated transcriptional repression of numerous cytokine and chemokine genes^23^. Moreover, the conditional deletion of GRIP1 in macrophages sensitized mice to LPS-induced endotoxin shock^23^ suggesting that GRIP1 is protective against systemic inflammation *in vivo* due to its unexpected inhibitory role in inflammatory macrophage activation. In contrast, the mechanisms of GC-induced homeostatic macrophage polarization *in vitro* or *in vivo* are not well described. Interestingly, we showed recently that *Klf4* in bone marrow-derived macrophages (BMDM) is strongly induced by GCs^24^ suggesting that GR may promote homeostatic macrophage phenotype by inducing *Klf4* expression and, potentially, GRIP1 may contribute to this process by serving as a conventional GR coactivator.

Here, we report that GRIP1 promotes the induction of anti-inflammatory macrophages in a manner that is independent of its function as GR coregulator. We present data on the molecular mechanisms underlying this unexpected role of GRIP1 in primary macrophages and *in vivo*. Further, we report that a genetic conditional deletion of GRIP1 in macrophages *in vivo* impairs not only the homeostatic control of inflammatory macrophages, but also the maintenance of the tissue-resident macrophages resulting in a multi-symptom metabolic disease in the context of an obesity-induced model of metabolic challenge.

## Results

### GRIP1 is required for efficient macrophage activation by IL4

**Figure 1:**
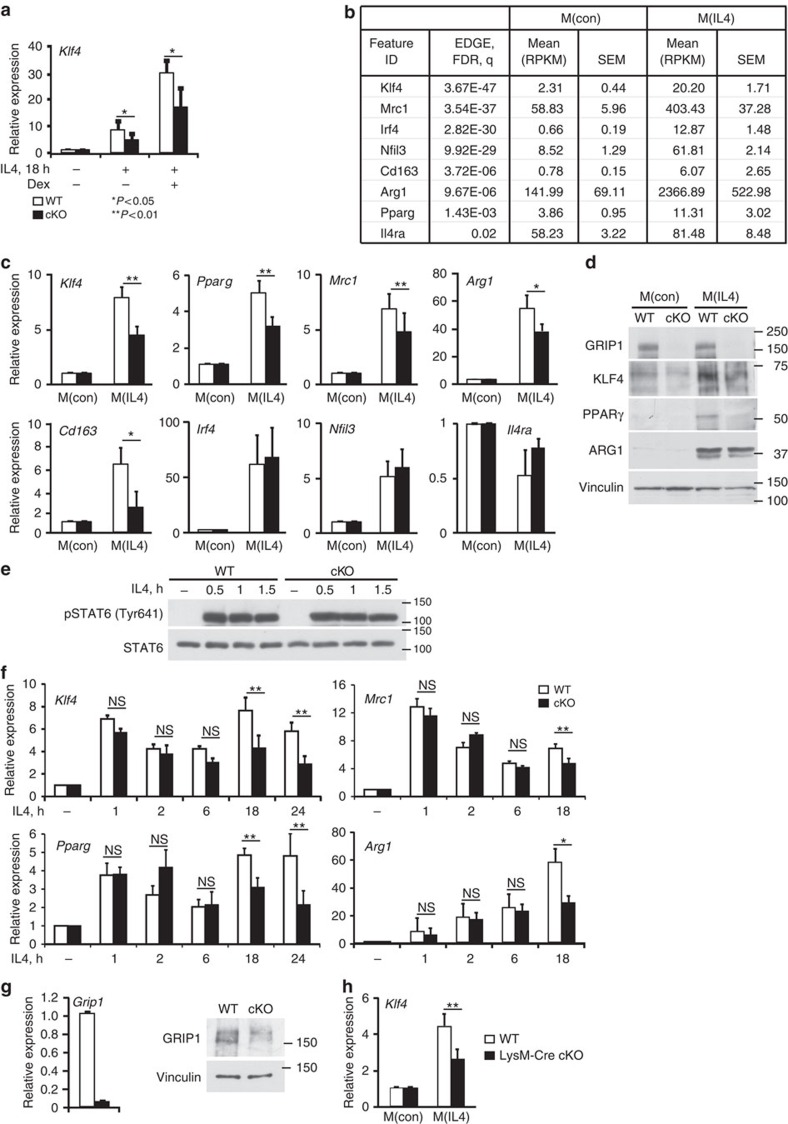
GRIP1 is required for M(IL4) polarization. (**a**) GRIP1 deletion attenuates *Klf4* mRNA induction during IL4-induced macrophage activation. WT and cKO BMDM were activated with IL4 (20 ng ml^−1^, 18 h) followed by treatment with Dex (100 nM, 30 min) and *Klf4* expression was assessed by RT-qPCR using β-actin as an internal normalization control. *Klf4* transcript levels in BMDM not exposed to IL4 were set to 1 for each genotype; *n*=3/group, error bars are s.d. Student's *t*-test was used to determine significance. (**b**) A subset of genes differentially expressed in unstimulated [M(con)] versus IL4-activated [M(IL4)] BMDM as detected by RNAseq. (**c**) Lack of GRIP1 impairs the expression of a subset M(IL4) polarization-associated genes. Gene expression was assessed by RT-qPCR as in **a** and compared using the Mann–Whitney test. *n*>6 per group; error bars are s.e.m. (**d**) GRIP1-deficient M(IL4) express less KLF4, PPARγ and ARG1 proteins. M(con) and M(IL4) WCE were analysed for the expression of indicated proteins by immunoblotting with vinculin as a loading control. Shown is a representative of 2–3 blots (see Supplementary Fig. 1 for quantitation of multiple experiments and a full size KLF4 blot). (**e**,**f**) IL4-mediated STAT6 activation is intact in GRIP1 cKO macrophages. WT and cKO BMDM were incubated with IL4 for indicated times and the level of total and Tyr641-phosphorylated STAT6 in WCE was assessed by immunoblotting (**e**). STAT6 target gene expression in WT and cKO macrophages incubated with IL4 for indicated times was assessed by RT-qPCR as in **a**. *n*=3/group; error bars are s.e.m. (**f**). (**g**,**h**) The importance of GRIP1 for *Klf4* induction is mouse strain-independent. GRIP1 LysM-Cre cKO and matching WT mice were produced as described in Methods section and Supplementary Fig. 3. M(con) and M(IL4) were generated and the expression of *Grip1* (**g**) and *Klf4* (**h**) were assessed by RT-qPCR as in **a**; normalized *Grip1* transcript level is set to 1 in WT M(con); normalized *Klf4* transcript level is set to 1 in M(con) of each genotype; *n*=4/group; error bars are s.d. Student's *t*-test was used to determine significance. GRIP1 protein expression in WT and GRIP1 LysM-Cre cKO M(con) was assessed by immunoblotting with vinculin as a loading control.

To investigate whether GRIP1 is involved in GR-mediated induction of *Klf4*, we first evaluated by quantitative PCR with reverse transcription (RT-qPCR) *Klf4* expression in BMDM from Mx1-Cre^−/−^; GRIP1^fl/fl^ (wild type (WT)) and Mx1-Cre^+/+^; GRIP1^fl/fl^ (conditional knockout (cKO)) mice^23^. Following an 18-h activation of BMDM with IL4, we pulsed the resulting M(IL4) with synthetic GC Dexamethasone (Dex) and found the induction of *Klf4* in GRIP1-deficient macrophages to be modestly attenuated (Fig. 1a). Unexpectedly, relative to WT M2(IL4), the expression of *Klf4* was significantly reduced in cKO immediately following IL4-mediated activation (Fig. 1a) suggesting a role of GRIP1 in *Klf4* induction that is GC- and, thus, GR-independent. Using genome-wide transcriptome analysis of untreated BMDM [M(con)] and differentiated M(IL4), we identified 1211 differentially expressed genes (false discovery rate (FDR) corrected *P*<0.01) and selected for further study genes strongly upregulated in M(IL4) (Fig. 1b) consistent with previous results^25^,^26^. As expected, *Klf4* was strongly induced along with a number of other M(IL4)-signature genes (*P*=3.67E-47 (EDGE R); Fig. 1b). Given that KLF4 is a ‘master regulator' of IL4-induced polarization and drives the expression of many M2-enriched genes, we compared the expression of such transcripts in WT and cKO macrophages: similar to *Klf4*, *Pparg*, *Mrc1*, *Arginase1* (*Arg1*) and *Cd163* were expressed at comparable levels in naïve WT and GRIP1 cKO M(con) but considerably less induced by IL4 in absence of GRIP1 (Fig. 1c). Furthermore, KLF4 and PPARγ protein levels were also significantly reduced in the cKO M(IL4) (Fig. 1d and Supplementary Fig. 1) corroborating the RNA expression data. Interestingly, the impaired induction of the M(IL4) genes was not uniform as the expression of other genes reportedly typical of the homeostatic macrophage phenotype, for example, interferon regulatory factor 4 (*Irf4*)^27^, NF IL3-regulated (*Nfil3*)^28^ and Il4 receptor alpha (*Il4ra*)^29^, was unaffected by GRIP1 deletion (Fig. 1c).

Because IL4 signals through STAT6 to alter gene expression^13^,^29^,^30^, we assessed whether the IL4 signalling pathway was intact in GRIP1 cKO macrophages. Figure 1e demonstrates that WT and cKO macrophages show equal expression of STAT6 as well as comparable STAT6 phosphorylation on activating Tyr641 in response to IL4 stimulation. Consistently, the induction of typical M(IL4) genes that are direct targets of STAT6 in GRIP1 cKO was similar to WT at early time points (up to 6 h) of IL4 treatment (Fig. 1f and Supplementary Fig. 2) but dropped progressively by 18–24 h (Fig. 1f). Collectively, these results suggest that GRIP1 facilitates the IL4-induced differentiation of M(IL4) at later time points by a mechanism that operates downstream of STAT6 activation.

To verify that the consequences of GRIP1 loss on M(IL4) polarization were strain-independent, we have created a new mouse strain, the LysM-Cre GRIP1 cKO, (Methods section and Supplementary Fig. 3a) in which GRIP1 was deleted specifically in the myeloid lineage. In BMDM from these mice, we observed a near complete loss of GRIP1 transcript and protein (Fig. 1g), whereas the expression of two other p160 family members, *Src1* and *Src3*, did not substantially differ between genotypes (Supplementary Fig. 3b). Importantly, when these BMDM were polarized to the M(IL4) phenotype, the induction of *Klf4* was significantly attenuated relative to matching WT (Fig. 1h) suggesting that a requirement for GRIP1 in M(IL4) differentiation is mouse strain-independent.

### GRIP1 serves as a KLF4 coactivator

Lack of GRIP1 led to an impaired induction of several known KLF4 target genes (for example, *Arg1*, *Mrc1*, *Pparg* and *Klf4* itself). Importantly, this defect was only apparent following a long-term macrophage exposure to IL4, that is, conditions under which the KLF4 protein, whose expression is initially activated by STAT6, accumulates in a cell to further enforce the M(IL4) cell fate commitment. Therefore, we reasoned that, perhaps, GRIP1 functions as a KLF4 coactivator directly participating in the transcriptional regulation of KLF4 target genes.

**Figure 2:**
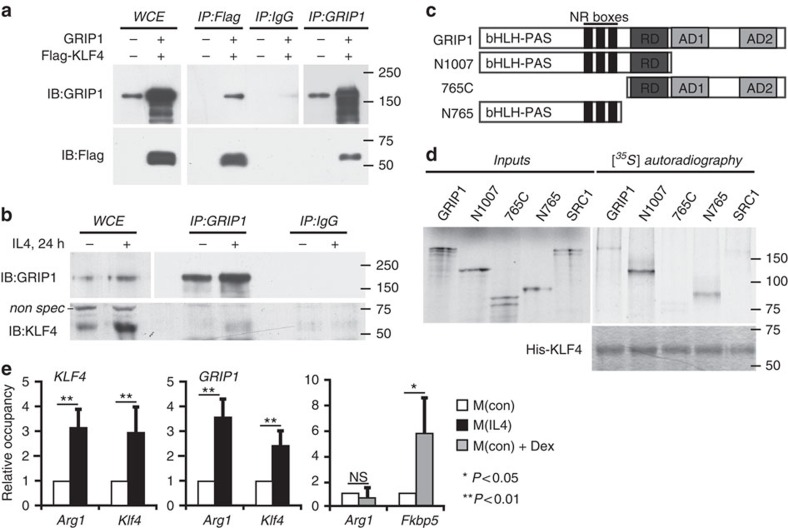
GRIP1 interacts with KLF4 *in vivo* and *in vitro.* (**a**) GRIP1 and Flag-tagged KLF4 were overexpressed in 293T cells, and WCE were immunoprecipitated (IP) with antibodies to Flag, GRIP1 or normal anti-rabbit IgG, as indicated. Proteins were detected by immunoblotting (IB) using anti-GRIP1 and anti-Flag antibodies. (**b**) Endogenous GRIP1 and KLF4 interact in J774A.1 cells. Cells were treated as shown and WCE were immunoprecipitated with anti-GRIP1 or anti-rabbit IgG. GRIP1 and KLF4 were detected by immunoblotting. To visualize GRIP1 in WCE, a longer exposure of the blot is shown. (**c**) Diagrammed are the full-length GRIP1 or truncated mutants used in the interaction assay. Basic HLH-PAS domain, repression domain (RD), AD1 and 2 and NR-interacting ‘boxes' are marked. (**d**) The N-terminal region of GRIP1 physically interacts with KLF4 *in vitro*. Indicated GRIP1 derivatives (**c**) and a full-length SRC1 were *in vitro* transcribed and translated in the presence of [^35^S]-methionine and incubated with the bacterially expressed full-length His-tagged KLF4 immobilized on metal-affinity resin. The top panel shows autoradiography of inputs, and bound derivatives; Coomassie blue stained His-KLF4 is shown at the bottom. (**e**) GRIP1 is recruited to KLF4 target genes in M(IL4). The recruitment of KLF4 and GRIP1 to KLF binding sites of *Arg1* and *Klf4* was assessed by ChIP in M(con) and M(IL4) as described in Methods section. As control (right panel), M(con) were treated with 100 nM Dex for 40 min, as indicated, and GRIP1 occupancy was evaluated by ChIP at the KLF4-binding site of *Arg1* or at the GRE of *Fkbp5*. For each site, shown are mean±s.d. of three or more independent experiments.

To explore this possibility, we first assessed whether GRIP1 and KLF4 are within the same protein complex. 293T cells were transfected with GRIP1- and Flag-tagged KLF4-expressing plasmids, whole-cell extracts (WCE) prepared and subjected to co-immunoprecipitation. As shown in Fig. 2a, pulling down Flag-KLF4 co-precipitated GRIP1 and vice versa, whereas neither protein was precipitated with normal rabbit IgG control antibodies. To test for the interaction between endogenous GRIP1 and KLF4 in macrophage-like cells, WCE of J774A.1 mouse monocytic cells (untreated or treated with IL4 for 24 h to induce KLF4 expression) were immunoprecipitated with anti-GRIP1 antibody or IgG as control, and blotted for KLF4. As shown in Fig. 2b, KLF4 protein level was indeed significantly higher in IL4-treated cells, and GRIP1 co-precipitated KLF4 under these conditions.

To test whether there is a direct physical interaction between GRIP1 and KLF4, we performed *in vitro* pull-down experiments with purified proteins. Full-length GRIP1 (diagrammed in Fig. 2c) or, as control, another structurally similar member of the p160 family, SRC1, were *in vitro* transcribed and translated and tested for their ability to bind His-tagged recombinant KLF4 immobilized on an affinity matrix. As shown in Fig. 2d, GRIP1 but not SRC1 were retained by His-KLF4. GRIP1 is a modular protein with well-defined protein–protein interaction domains: the N-terminal bHLH-PAS domain, the centrally located NR boxes responsible for NR binding, the C-terminal AD1 and AD2 that recruit histone modifiers and a repression domain which mediates GRIP1 ability to serve as a GR corepressor (Fig. 2c). To begin to evaluate the importance of individual GRIP1 regions in mediating the interaction with KLF4, we tested GRIP1 truncation mutants lacking a part or all of its C-terminal half including its transcriptional regulatory domains (N1007 and N765, respectively) or the N-terminal half including the bHLH/PAS domain (765C; Fig. 2c). We found that both N1007 and N765 bound KLF4, whereas 765C did not (Fig. 2d) suggesting that GRIP1:KLF4 interaction is mediated by the N-terminal portion of GRIP1.

Our protein–protein interaction data, together with the functional studies pointing to the importance of GRIP1 in KLF4-driven gene expression, were consistent with an idea that GRIP1 may act as a KLF4 coactivator at a subset of KLF4 target genes in M(IL4). Our chromatin immunoprecipitation (ChIP) assays showed expected KLF4 recruitment to the known KLF binding sites of the *Klf4* and *Arg1* genes in differentiated M(IL4) (Fig. 2e). Strikingly, GRIP1 was co-recruited to the same KLF binding sites as KLF4 under IL4-polarizing conditions. As a specificity control, we treated M(con) with Dex, and found no GRIP1 occupancy above baseline at the KLF4 target *Arg1*, whereas the GRE of the classic GR target *Fkbp5* showed a dramatic Dex-dependent GRIP1 enrichment (Fig. 2e). Collectively, these results demonstrate that GRIP1 is recruited to KLF4 target genes in M(IL4) consistent with a direct participation in the transcriptional programming of IL4-activated macrophages.

### WAT inflammation and MΦ phenotype switch in obese cKO

**Figure 3:**
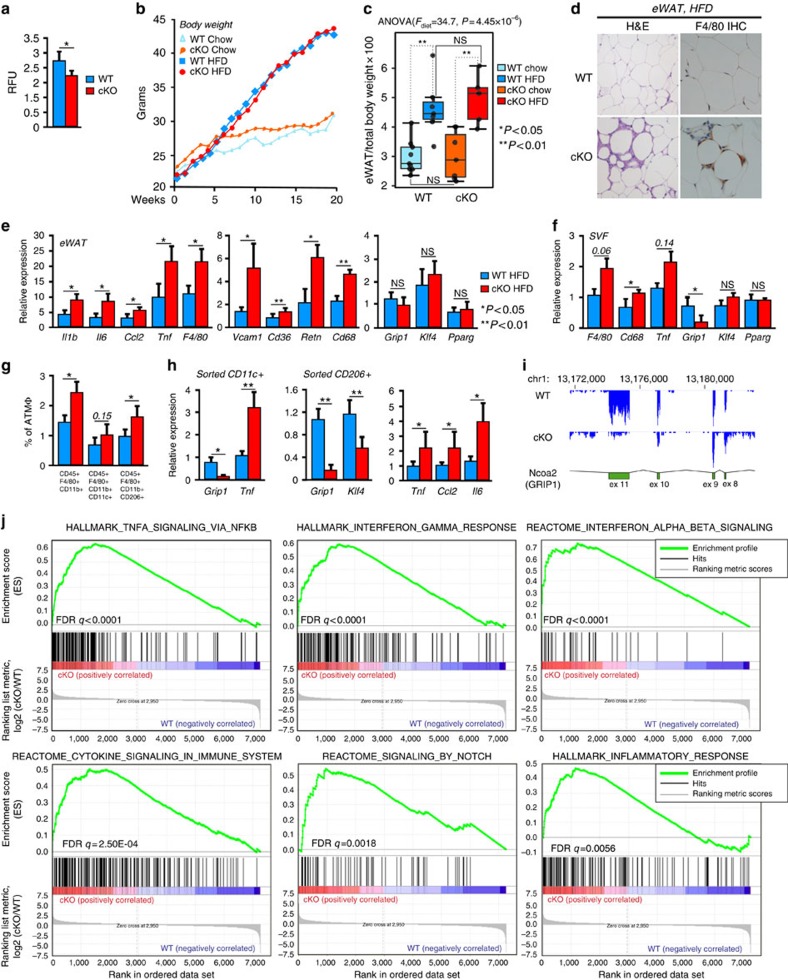
GRIP1 deficiency in macrophages promotes HFD-induced inflammation in WAT *in vivo.* (**a**) FA uptake is impaired in GRIP1-deficient M(IL4). FA uptake was measured 6 min post-FA addition and expressed in relative fluorescence units (RFU). *n*>5 per group; error bars are s.d.; Student's *t*-test was used to determine significance. (**b**) WT and GRIP1 cKO gain similar amount of body weight. Shown are averages of 9 mice per group for chow-fed mice and 7 WT and 5 cKO mice per group for HFD-fed mice. (**c**) eWAT weight in WT and cKO after 20 weeks of chow or HFD. eWAT weight is expressed as percentage of total body weight for each mouse; *n*>10 per group. Significance was determined using ANOVA followed by Tukey's HSD test. The *F*-statistic and the *P* value for the significant main effect are shown (Methods section, Supplementary Fig. 4a and Supplementary Table 1 for detailed statistics). (**d**) Haematopoietic cell infiltration in cKO eWAT following HFD. eWAT was fixed in 4% PFA, paraffin-embedded and subjected to H&E staining or F4/80 immunohistochemistry. Magnification is × 40. (**e**) Augmented expression of inflammatory mediators in eWAT of the HFD-fed GRIP1 cKO. Gene expression was assessed by RT-qPCR with *Hprt* for normalization. *n*>5 per group; error bars are s.d. The Mann–Whitney test was used to determine significance. (**f**) SVF cells from HFD-fed GRIP1 cKO show increased expression of macrophage and inflammatory markers. Gene expression in WT and cKO SVF was assessed as in **e**. *n*=3/group; error bars are s.d. (**g**) GRIP1 cKO SVF shows increased macrophage abundance. The percentage of CD45+F4/80+CD11b+ macrophages in the SVF of WT and cKO mice and frequencies of the sorted CD11c+ (CD45+F4/80+CD11b+CD206-) and CD206+ (CD45+F4/80+CD11b+CD11c-) macrophages were quantified by FACS using FlowJo software. *n*=5/group; values are mean±s.e.m. The Mann–Whitney test was used to determine significance. (**h**) GRIP1 cKO ATMΦ display an exaggerated inflammatory profile. The expression of indicated genes in CD11c+ and CD206+ populations from **g** was assessed as in **e**. *n*>4 per group; error bars are s.d. Student's *t*-test was used to determine significance. (**i**) Efficient deletion of *Ncoa2* (GRIP1) in CD11c+ macrophages of cKO mice. RNAseq shows read distribution across *Ncoa2* exons 8–11 and a complete deletion of Ex11. (**j**) GSEA in WAT-derived CD11c+ macrophages shows upregulation in cKO versus WT of multiple gene signatures related to inflammatory response and interferon signalling. Shown are top gene enrichment profiles with corresponding FDR q.

Tissue-resident macrophages contribute to the regulation of lipid homeostasis in part by carrying out fatty acid (FA) uptake and β-oxidation in tissues^31^. FA uptake is reportedly defective in M(IL4) lacking KLF4 (ref. 14) but not in macrophages lacking other transcription factors such as PPARγ also implicated in M(IL4) differentiation^15^. Because GRIP1 was an important contributor to KLF4 target gene induction including the *Klf4* gene itself and, consequently, M(IL4) activation, we tested whether a decreased level of KLF4 affects a metabolic function of GRIP1-deficient macrophages. Figure 3a shows that indeed FA uptake by the cKO M(IL4) *in vitro* was significantly attenuated compared with WT.

The balance between monocyte-derived infiltrating and tissue-resident macrophages in metabolic tissues is a key contributing factor in the maintenance of metabolic health. Because loss of GRIP1 impaired M(IL4) polarization and function *in vitro*, we evaluated the role of myeloid GRIP1 in the systemic metabolic homeostasis in a diet-induced obesity model of insulin resistance. We placed 8-week-old WT and GRIP1 cKO mice on high-fat diet (HFD) or regular chow, as control, and evaluated their phenotype 20 weeks later. As shown in Fig. 3b, although HFD-fed mice developed striking obesity, there was no difference in body weight increase between WT and cKO either on regular chow or HFD. Consistently, the weights of epididymal fat pads (eWAT) isolated from WT and GRIP1 cKO mice after 20 weeks of HFD were ∼2-fold higher than those from chow-fed animals, but displayed no differences between the two genotypes (Fig. 3c, Supplementary Fig. 4a and Methods section).

A prolonged exposure to HFD leads to monocyte recruitment and differentiation into macrophages in WAT, sustained production of inflammatory cytokines and chemokines and chronic inflammation that has been postulated to promote WAT dysfunction, insulin resistance and T2DM in several models of obesity^6^,^32^. Given our earlier data on the increased sensitivity of GRIP1 cKO mice to a systemic acute inflammatory challenge^23^, we performed a histological analysis of the eWAT of these mice. The haematoxylin and eosin (H&E) staining of the eWAT following 20 weeks of HFD showed disrupted adipocyte morphology and a striking increase in the number of infiltrating hematopoietic cells in GRIP1 cKO compared with WT mice (Fig. 3d). Furthermore, the immunohistochemical analysis of the eWAT for the macrophage surface marker F4/80 confirmed that many infiltrating cells in GRIP1 cKO are macrophages (Fig. 3d).

Corroborating histological findings, gene expression profiling in total eWAT RNA from the HFD-fed cKO mice revealed adipose tissue inflammation, during which adipocytes actively produce inflammatory cytokines and chemokines perpetuating additional immune cell migration and activation. Figure 3e shows a significant increase, relative to WT, in the transcript levels of macrophage markers (F4/80 and Cd68) and inflammatory mediators (*Il1b, Il6, Ccl2, Tnf, Vcam1* and *Retn*). Among these, TNF, IL6, CCL2 and RETN (known as resistin, an adipocyte-produced peptide) have been directly implicated in the pathogenesis of insulin resistance^33^,^34^,^35^,^36^,^37^. Notably, in total eWAT composed of predominantly adipocytes, the expression of *Grip1* itself, as well as *Klf4* and *Pparγ*—critical factors for adipocyte differentiation^38^,^39^,^40^—was, as expected, similar in the WT and cKO (Fig. 3e), consistent with the lack of GRIP1 deletion in these cells. Of note, no adverse phenotype of macrophage GRIP1 deletion was seen in the lean eWAT of chow-fed mice of the same age (Supplementary Fig. 5a,b).

To dissociate the effects of GRIP1 deficiency in ATMΦ from those on other cell types, we first isolated the total stromal vascular fraction (SVF) from eWAT of HFD- and chow-fed WT and cKO. In the resulting cell mix, we still detected an increase in the expression of macrophage markers (*F4/80* and *Cd68*) and *Tnf* in the GRIP1 cKO mice compared with WT in HFD- but not chow-fed mice and, importantly, GRIP1 depletion due to its deletion in myeloid cells has become apparent (Fig. 3f and Supplementary Fig. 5c). Next, we quantified ATMΦ in SVF of HFD-fed mice by FACS based on their CD45+CD11b+F4/80+ phenotype^41^; consistent with results of immunohistochemistry (Fig. 3d), GRIP1 cKO mice had considerably higher percentage of ATMΦ in SVF compared with WT littermates (Fig. 3g).

To better understand which cells are responsible for WAT inflammation observed in GRIP1 cKO mice, we purified by FACS the two ATMΦ subpopulations, the inflammatory CD11c+ and homeostatic resident CD206+. Quantification of FACS data revealed a significant increase in the GRIP1 cKO CD206+ resident population relative to that in WT; CD11c+ cell frequency was also trending higher in the cKO (Fig. 3g), even though this result is an underestimate of the CD11c+ cell accumulation because lipid-filled foam cells derived from inflammatory CD11c+ ATMΦ are expected to be lost in the lipid fraction during SVF purification. The analysis of total RNA from both populations showed a near complete loss of *Grip1* confirming the efficiency of deletion *in vivo* (Fig. 3h). The expression of *Tnf* in GRIP1 cKO infiltrating CD11c+ was dramatically increased relative to WT (Fig. 3h) in line with an increase observed in total eWAT. Interestingly, GRIP1 cKO tissue-resident CD206+ macrophages also produced more *Tnf*, *Ccl2* and *Il6* and, strikingly, less *Klf4* compared with WT (Fig. 3h) recapitulating results seen in the *in vitro* activated M(IL4) (Fig. 1a-c). Thus, under conditions of HFD, GRIP1-deficient tissue-resident anti-inflammatory macrophages were undergoing a phenotypic switch *in vivo* towards a more inflammatory cell type.

To better gauge the extent of inflammatory response that was occurring in the WAT of the cKO, we used low-yield RNAseq protocol (Methods section) to evaluate CD11c+ macrophage transcriptome in the remaining RNA from WT and cKO infiltrating macrophages. Read distribution near GRIP1 Ex10 and 11 (Fig. 3i) confirmed near complete deletion of GRIP1 Ex11 consistent with the originally reported GRIP1 whole-body KO (ref. 42). We performed gene set enrichment analysis (GSEA) to compare expression profiles of the 7,260 identified genes in the WT and cKO CD11c+ macrophages and identify specific affected sets. This analysis revealed a remarkable upregulation of genes associated with inflammation and interferon response in GRIP1 cKO macrophages. The top three enriched sets (FDR, *q*<0.0001) included TNF signaling via NFkB (MSigDB, M5890), interferon gamma response (M5913) and interferon alpha/beta signaling (M973) followed by several gene sets enriched in inflammation and inflammatory disorders (Fig. 3j, Supplementary Data 1). Only few enriched gene sets among top 50 with FDR *q*<0.1 were not directly linked to inflammation indicating that GRIP1-deficient eWAT infiltrating macrophages are not only present in greater numbers but are also more activated relative to their WT counterparts, consistent with the previously described role of GRIP1 as an inhibitor of inflammatory response^23^.

### Obese GRIP1 cKO mice develop liver inflammation and steatosis

Prolonged HFD exposure in humans induces liver steatosis, a manifestation of NAFLD that is frequently associated with obesity and insulin resistance^43^. The pathogenesis of NAFLD is driven by activation of the liver-resident Kupffer cells and further recruitment of inflammatory cells from the circulation^44^.

**Figure 4:**
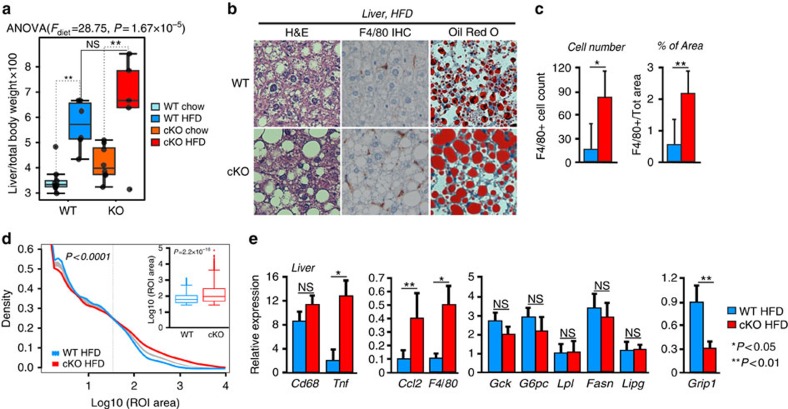
GRIP1 cKO HFD-fed mice develop liver inflammation and steatosis. (**a**) Liver weight in WT and cKO mice after 20 weeks of chow or HFD. All liver weights are expressed as per cent of total body weight; *n*>10 per group. The statistical significance of differences was determined using ANOVA following Tukey's HSD test. The *F*-statistic and the *P* value for the significant main effect are shown above the panel (see Methods section, Supplementary Fig. 4b and Supplementary Table 2 for detailed statistics). (**b**) Haematopoietic cell infiltration and lipid accumulation in liver following HFD. Livers were processed as in Fig. 3c for H&E staining or F4/80 immunohistochemistry, or frozen and stained with Oil Red-O to visualize neutral lipids. Magnification is × 20. (**c**) Quantification of macrophage infiltration. F4/80-stained sections from **b** of 5 WT and 6 cKO livers were scanned and analysed using ImageJ Color Deconvolution 1.5 plugin as described in Methods section. The average number of F4/80-stained cells and the percentage of F4/80-stained area of the image in WT versus cKO were compared by the Mann–Whitney test. (**d**) Quantitative analysis of fat droplet sizes from **b** in WT and GRIP1 cKO HFD-fed mice (*n*=5 each). The area of fat droplets (ROI area) was determined using ImageJ (Methods section). KDEs of the lipid droplet size distributions (ncKO=40,110, nWT=9,985) were constructed and compared using *sm* (R) as described in Methods. Permutation test (*N*=10,000) was performed to test for the equality of cKO and WT KDE (*P*<0.0001); the 95% confidence interval for the equality of distributions is shown in grey. To demonstrate the differences in the frequency of larger droplets, the inset shows the Mann–Whitney comparison of droplet sizes with the area above 28 pixels. (**e**) The expression of inflammatory mediators, but not genes involved in glucose and fat metabolism, is deregulated by GRIP1 deletion in livers of HFD-fed mice. Gene expression was assessed by RT-qPCR with *Hprt* as a normalization control. *n*>5 per group; error bars are s.d. The statistical significance of differences was calculated using the Mann–Whitney test.

Similar to eWAT, normalized weights of livers isolated from WT and GRIP1 cKO mice after 20 weeks of HFD were significantly higher than those from chow-fed animals, but displayed no significant differences between the two genotypes (Fig. 4a, Supplementary Fig. 4b and Methods section). As with eWAT, immunohistochemistry and quantitative image analysis (Methods section) revealed more F4/80+ cells in GRIP1 cKO livers compared with WT (Fig. 4b,c) which was corroborated by the elevated level of F4/80 transcript (Fig. 4e). Importantly, despite lack of significant differences in liver weights between WT and cKO HFD-fed mice (Fig. 4a), a dramatic change in the overall liver morphology with disrupted architecture of hepatocytes and accumulation of large droplet-like structures was apparent in the cKO even following H&E staining or F4/80 immunohistochemistry (Fig. 4b). Indeed, the Oil red-O staining of neutral lipids in frozen liver sections from cKO mice revealed striking lipid accumulation compared with WT (Fig. 4b). This was further supported by the quantitative analysis of the size distribution of lipid droplets in Oil red-O-stained liver slices (Methods section), which confirmed a predominance of large lipid droplets in cKO relative to WT consistent with ‘fatty liver'-like pathology (Fig. 4d). As expected, gene expression analysis showed no increase in the abundance of CD68 considered to be a marker of Kupffer cells^45^, whereas the levels of inflammatory mediators (*Tnf* and *Ccl2*) were augmented in cKO livers (Fig. 4e). Interestingly, despite a substantial deletion of GRIP1 in hepatocytes of cKO, the expression of genes involved in glucose and fat metabolism (*Gck*, *G6p*, *Lpl*, *Fasn* and *Lipg*) remained unaffected (Fig. 4e) supporting the idea that the metabolic phenotype of GRIP1 cKO mice is due to the exacerbated HFD-induced chronic inflammation. Of note, liver morphology and the expression of inflammatory mediators and metabolic enzymes in livers of age-matched chow-fed mice was comparable between the two genotypes (Supplementary Fig. 5d,e) suggesting that the inflammatory status of GRIP1 cKO within the time-frame examined is driven by the HFD. Taken together, these results show that GRIP1 deletion leads to hepatic inflammation and ‘fatty liver' during HFD challenge.

### GRIP1 cKO HFD-fed mice develop glucose intolerance

**Figure 5:**
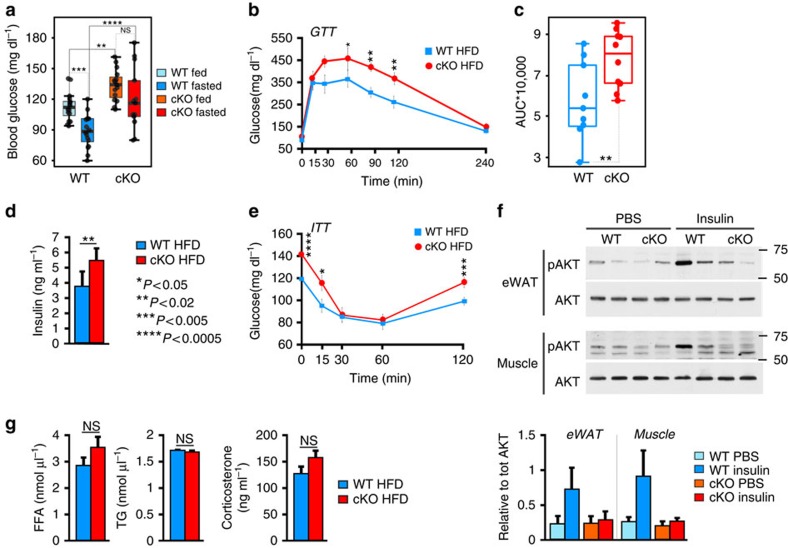
GRIP1 cKO HFD-fed mice develop glucose intolerance. (**a**) Blood glucose levels in WT and cKO during *ad libitum* HFD feeding or after an overnight starvation. *n*>9 per group. The differences between fed and fasted mice within and between genotypes were evaluated by mixed linear modelling (Methods section and Supplementary Fig. 4c,d). The statistical significance of pairwise comparison of means was evaluated using Tukey's test with Holm's corrections for multiple comparisons (Methods section and Supplementary Tables 3–5). (**b**) GTT in HFD-fed WT (*n*=10) and cKO (*n*=11). Overnight-starved HFD-fed mice were injected with glucose (1.5 mg g^−1^ body weight, intraperitoneally (IP)) and blood glucose was measured at indicated times. GTT was analysed using mixed linear modelling (Methods section). Error bars are s.e.m. (**c**) The areas under the GTT curves (AUC) for individual animals were calculated using trapezoid methods in R and the Mann–Whitney test was used to determine significance. (**d**) Serum insulin levels were measured by ELISA in WT and cKO after 20 weeks of HFD. *n*>5 per group; error bars are s.d. The Mann–Whitney test was used to determine significance. (**e**) Insulin tolerance test (ITT) in HFD-fed WT and cKO mice. Mice were fasted for 4 h and injected with insulin (0.75 U kg^−1^ body weight, IP). Blood glucose levels were measured before (time 0) and at indicated times post-injection. *n*=13 and 15 for WT and cKO, respectively, except at 15 min when *n*=5 for each genotype; error bars are s.e.m. The Mann–Whitney test was used to determine significance. (**f**) HFD-fed GRIP1 cKO mice display insulin resistance in eWAT and muscle. WT and cKO mice were injected with PBS or insulin (0.75 U kg^−1^ body weight, IP), as indicated, killed 10 min later and WCE prepared from eWAT and muscle. The level of total and Thr308-phosphorylated Akt in WCE was assessed by immunoblotting. Blots for 2 mice per group were quantified using ImageJ. pAkt signals were normalized to those of total Akt; error bars are s.e.m. (**g**) Serum levels of FFA (*n*>5 each genotype), triglycerides (TG; *n*=12 each) and corticosterone (*n*=6 each) in HFD-fed WT and cKO were compared using Student's *t*-test. Error bars are s.e.m.

One of the consequences of prolonged HFD feeding is the development of obesity-related insulin resistance which stems from a combination of altered function of insulin target cells and the accumulation of inflammatory macrophages in WAT (refs 3, 32). To assess the metabolic status of our HFD-fed mice, we monitored glucose levels in WT and GRIP1 cKO mice during *ad libitum* feeding and after overnight starvation. WT mice displayed normal blood glucose levels which dropped following overnight fasting (Fig. 5a, Supplementary Fig. 4c,d, Methods section and Supplementary Tables 3–5). In contrast, GRIP1 cKO mice were hyperglycemic at baseline and, strikingly, displayed no apparent response to overnight starvation (Fig. 5a, Supplementary Fig. 4c-d, Methods section and Supplementary Tables 3–5). To determine whether GRIP1 deficiency results in impaired ability to lower the blood glucose level, we performed a glucose tolerance test (GTT). As shown in Fig. 5b,c, GRIP1 cKO mice had significantly elevated glucose levels after challenge relative to WT over the course of several hours. Moreover, serum insulin levels in HFD-fed GRIP1 cKO mice were also significantly increased compared with those in WT (Fig. 5d). In an insulin tolerance test, injection of hyperglycemic cKO mice led to an eventual drop of glucose levels down to those seen in WT, but immediate reversal back to hyperglycaemia (Fig. 5e). Thus, at 20 week of HFD, cKO were still transiently responsive to exogenously provided insulin, but endogenous elevated levels of insulin were insufficient to counteract hyperglycaemia. To assess whether WT and cKO mice display a difference in response to acute insulin administration in metabolic tissues, we injected HFD-fed mice with insulin and harvested their eWAT and muscle 10 min later to evaluate the Thr308 phosphorylation of Akt—a downstream kinase in the insulin signalling pathway^46^. As shown in Fig. 5f, there was no detectable difference in Akt phosphorylation between genotypes in control (PBS-injected) mice despite baseline hyperinsulinemia of GRIP1 cKO, and insulin-injected WT mice displayed a significantly higher level of pAkt in eWAT and muscle. Strikingly, no apparent difference in Akt phosphorylation was seen in insulin-injected cKO relative to control mice, consistent with insulin resistance of these tissues. In contrast, at 20-week time point, no significant difference in plasma levels of free FA (FFA), triglycerides and corticosterone—an endogenous GC that is known to promote insulin resistance—were seen in WT versus cKO mice (Fig. 5g).

As expected, WT and cKO mice fed normal chow showed similar fed and fasted blood glucose levels, identical glucose tolerance and comparable serum levels of insulin, FFA and triglycerides (Supplementary Fig. 6, Methods section and Supplementary Tables 3–5). Thus, the lack of GRIP1 in macrophages sensitizes mice to metabolic challenge resulting in chronic inflammation of metabolic tissues, hyperinsulinemia and glucose intolerance under HFD conditions.

## Discussion

Obesity establishes a state of low-grade inflammation in different tissues including WAT, vascular endothelium and liver, and is associated with the pathogenesis of T2DM, cardiovascular disease and NAFLD. Over time, obesity induces a massive infiltration of inflammatory macrophages in WAT in both humans and mice, and ATMΦ content correlates with levels of adiposity and insulin resistance^6^. These cells are the main source of local inflammatory mediators such as TNF, IL6 and CCL2; in fact, the disruption of genes encoding these cytokines in mice reduces macrophage infiltration of WAT and improves insulin sensitivity in obese animals^33^,^34^,^35^,^36^.

Notably, in obesity and other insulin-resistant conditions, ATMΦ encompass cells of different origin whose phenotypes span a spectrum of pro- and anti-inflammatory properties^9^. A key event in the induction of obesity-associated WAT inflammation and development of insulin resistance appears to be a shift in the overall balance of macrophages from the homeostatic tissue-resident phenotype to an inflammatory one^47^,^48^.

Despite the extreme plasticity of myeloid cells^10^,^49^ whose phenotypes are shaped by acute exposures to activating signals, distinct developmental origins and tissue-specific environments in which a given population resides^17^,^50^,^51^; the molecular characterization of transcription networks that specify macrophage function have focused primarily on inflammatory macrophages^52^,^53^,^54^,^55^,^56^. In contrast, the complex biology behind different populations of tissue-resident macrophages, in part due to their diversity, remains much less defined. Here we describe the function of a transcriptional coregulator which, in addition to its established role as a suppressor of inflammatory gene transcription, enables the optimal function of a key driver of the homeostatic macrophage phenotype.

Indeed, our *in vitro* and cell culture-based studies define GRIP1 as a classic KLF4 coactivator that physically binds KLF4 in cells and cell-free systems, is recruited in conjunction with KLF4 to KLF binding sites of known KLF4 target genes in M(IL4), and whose loss attenuates the expression of several genes known to be KLF4 targets, including, notably, the *Klf4* gene itself.

Interestingly, the absence of GRIP1 attenuated the expression of some M(IL4)-enriched genes but not others, and in our analysis thus far, only those genes that are reportedly direct targets of KLF4, rather than those of STAT6, were affected. This is also consistent with our data demonstrating that GRIP1 cKO macrophages retain normal IL4 signalling cascade and the induction of STAT6 target genes following brief IL4 exposure suggesting that both were GRIP1-independent.

KLF4 and other members of the KLF family of transcription factors are expressed in a highly tissue-selective manner and have been implicated in a variety of biological processes from reproduction and development to metabolism and regulation of cell death^57^. All KLFs share three C_2_H_2_-type Zn-finger motifs at their highly conserved C-terminal domains which are responsible for sequence-specific DNA binding^57^. Their N-terminal regions are variable in size and sequence and were used to classify KLF proteins into three subfamilies: KLFs 3, 8 and 12 (group 1) typically repress gene transcription by binding to the C-terminal binding proteins (CtBP); KLFs 1, 2, 4, 6 and 7 (group 2) share the N-terminal acidic ADs and typically act as transcriptional activators; KLFs 9, 10, 11, 13, 14 and 16 (group 3) can repress transcription through the N-terminal Sin3a-interacting domain but are also capable of acting as transactivators^57^,^58^. Further studies are needed to define the GRIP1-binding domain in KLF4, however, considering the high homology between group 2 KLFs, there is a formal possibility that GRIP1 interacts with other members of the KLF family acquiring a range of biological activities in a tissue-specific manner. KLF4 itself, in addition to its key role in macrophage activation, has been implicated in intestinal epithelial homeostasis acting as a tumour suppressor in colon cancer; furthermore, it promotes adipogenesis at early stages of adipocyte differentiation and is a critical factor in generating iPS cells^14^,^38^,^59^,^60^,^61^. It is tempting to speculate that the GRIP1:KLF4 interaction could potentially mediate KLF4 transcription programs in a cell- and context-specific manner.

**Figure 6:**
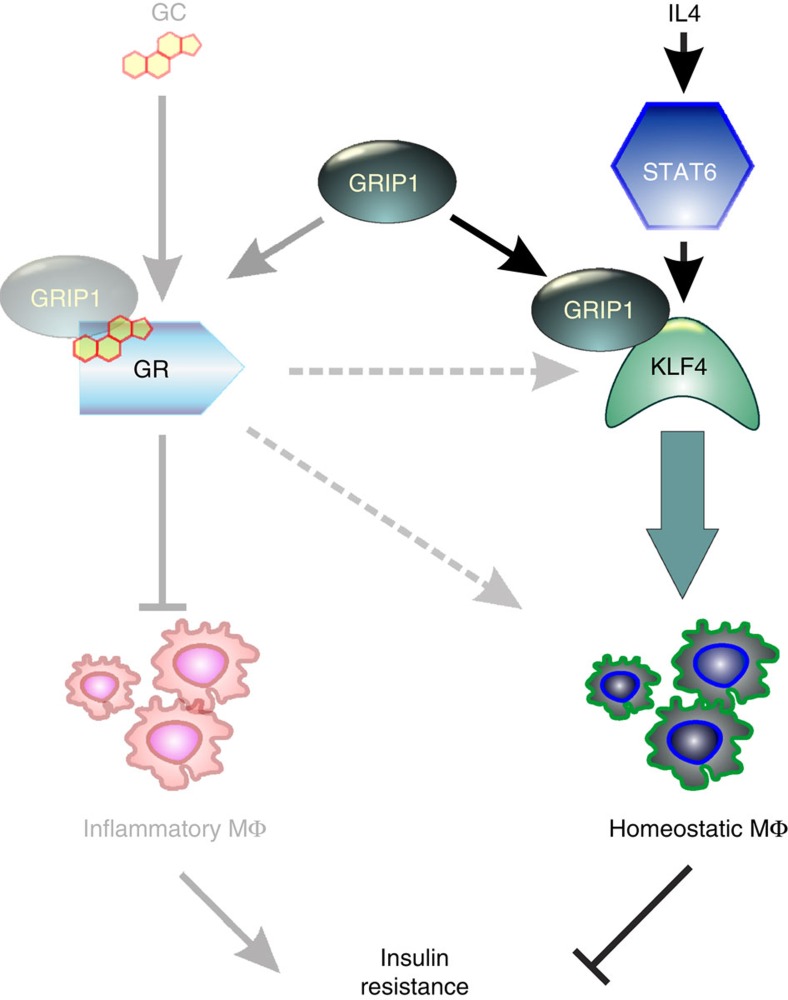
A model for GRIP1-dependent regulation of the balance between infiltrating and homeostatic macrophage populations. GRIP1 is recruited as a GR ligand-dependent corepressor to attenuate the transcription of pro-inflammatory mediators in infiltrating macrophages (bar-headed line) and as a KLF4 coactivator to facilitate the resident macrophage transcription programme (green arrow). In addition, GR activates the transcription of KLF4 (dashed arrow) and may have an as yet unidentified direct mechanism for facilitating tissue macrophage programming (dashed arrow). The two macrophage populations have opposing roles in metabolic homeostasis and insulin resistance.

Interestingly, GCs promote macrophage polarization programme *in vitro* resembling that elicited by IL4 (ref. 19). In that regard, it is noteworthy that KLF4, is strongly induced by GCs (refs 24, 62). Taking into consideration the established function of GRIP1 as a GR coactivator, our data are consistent with a model whereby pathways triggered by distinct stimuli functionally converge to initiate overlapping gene expression programs through a shared coregulator (Fig. 6; right). This model is further corroborated by colocalization of genomic GR and KLF binding sites, including those in the vicinity of the *Klf4* promoter^24^ and by an earlier observation that GR and KLF4 regulate partially overlapping sets of genes during epidermal barrier establishment in embryogenesis^63^. Furthermore, although the GRIP1 KLF4-binding region remains to be refined, GRIP1 may interact with GR (via NR box-3) and KLF4 (through the GRIP1 N-terminal region) simultaneously, perhaps within the same DNA:protein complex. This scenario appears plausible because *in vivo*, cells are likely to be exposed to IL4 and GCs at the same time, each signal promoting a specific part of the tissue-resident macrophage gene expression programme.

*In vivo*, GRIP1 deficiency in macrophages resulted in a metabolic syndrome-like disease when mice were challenged with HFD. Indeed, obese GRIP1 cKO mice displayed enhanced macrophage infiltration in eWAT and liver, increased production of cytokines and other inflammatory mediators in WAT and liver, and a general shift of homeostatic eWAT-resident macrophages towards more inflammatory phenotype as evidenced by enhanced expression of TNF, CCL2 and IL6 and blunted expression of KLF4. Importantly, consistent with loss of GRIP1 affecting both pro- and anti-inflammatory macrophage populations, our genome-wide transcriptome analysis of infiltrating monocyte-derived macrophages revealed their striking hyper-inflammatory phenotype whereby virtually every gene set over-represented in the cKO was linked to inflammation. We have previously identified GRIP1 as a corepressor for GR controlling the production of numerous inflammatory mediators^23^, hence, deregulated monocyte-derived macrophage activation in GRIP1 cKO is likely to result in part from the loss of this homeostatic control mechanism (Fig. 6, left).

This inflammatory state by 20 weeks of HFD resulted in the NAFLD-like changes in the liver with striking lipid accumulation, and, systemically, in fed and fasting hyperglycaemia, hyperinsulinemia and glucose intolerance all of which are indicative of developing insulin resistance. Indeed, although exogenous insulin transiently lowered blood glucose in GRIP1 cKO mice down to WT levels, the insulin signalling pathway in eWAT and muscle, as assessed by Akt phosphorylation, was no longer responsive, suggesting that these mice are likely to progress to a more severe resistance over time, similar to a human condition. We predict that in a time-frame of longer than 20 weeks of HFD used in this study, WT mice will ultimately succumb to glucose intolerance and steatotic liver, however, the absence of GRIP1 in myeloid cells dramatically accelerates this process.

To our knowledge, this is the first study to describe a direct role of a p160 coregulator in immunometabolism. GRIP1 was evaluated over a decade ago in total body KO mice which were reportedly protected from HFD-induced obesity, displayed reduced FA uptake and increased lipolysis in WAT, as well as enhanced adaptive thermogenesis in BAT. These effects were attributed to a possible role of GRIP1 in adipocytes as a coactivator for PPARγ (ref. 64). The apparent striking difference in the phenotype of the myeloid cell GRIP1 cKO is not entirely unexpected. In fact, the well-known diabetogenic systemic effects of GCs mediated by GR function in WAT and liver are in sharp contrast with the anti-inflammatory effects of GCs in macrophages. It is also notable that ‘fatty livers' in our GRIP1 cKO mice develop in the absence of appreciable change in the expression of liver metabolic enzymes. This phenotype is quite different from that caused by the hepatocyte-specific GRIP1 deletion which led to dramatic changes in liver enzyme expression on fasting^65^,^66^. These findings further underscore that in our GRIP1 cKO, the disease is caused primarily by macrophage-driven chronic inflammation rather than an intrinsic metabolic defect. They also suggest that future drug design efforts focused on tissue-selective therapeutics perhaps hold a greater promise than those aimed at systemic side effect-free compounds.

Collectively, our studies show the importance of GRIP1 in homeostatic macrophage activation and function. They suggest that GRIP1 relies on two distinct mechanisms in different macrophage populations to ensure metabolic health in face of HFD challenge (Fig. 6). Specifically, GRIP1 serves to restrain the transcription of inflammatory mediators by monocyte-derived macrophages and their infiltration of metabolic tissues, and, at the same time, it cooperates with GR, and more prominently, KLF4, to enforce the homeostatic transcription programme in tissue-resident macrophages. Given the importance of the immunomodulatory effects of tissue-resident myeloid cells in vascular homeostasis and wound healing, our data have broad implications for numerous inflammatory conditions beyond metabolic disease.

## Methods

### Statistical analysis for organ weight and blood glucose data

All data were presented as Mean±s.d. Continuous variables were compared using two-sided Student's *t*-test and Mann–Whitney *U* test.

Differences between organ weights were evaluated by two-way ANOVA with fat and liver weights normalized to total body weights as a response variable and type of diet (HFD versus CHOW) and animal genotype (WT versus cKO) as factors. Distribution of numerical data is shown via standard box-whisker plots (Figs 3c and 4a) in which the centre-line represents the median, the upper and lower-bounds of the box are 75 (Q3) and 25 (Q1) percentiles respectively, whereas the upper and lower whiskers are Q3+1.5 × IQR and Q1 – 1.5 × IQR, where IQR=Q3 – Q1. For the normalized eWAT weight, the between-group test indicates a single significant main effect of the diet type (Supplementary Table 1, bold; Supplementary Fig. 4a), but insignificant effect of the genotype and the lack of interactions between factors. Similarly, for the normalized liver weight, the between-group test indicates a single significant main effect of the diet type (Supplementary Table 2, bold; Supplementary Fig. 4b), but insignificant effect of the genotype and the lack of significant interactions between factors. Post hoc Tukey HSD test indicates the significance of HFD-CHOW differences for the WT and cKO mice (*P*=0.004 and *P*=0.005, respectively).

The differences between glucose levels were analysed using mixed linear modelling with the blood glucose level as a response variable, two between factors (diet and genotype) and one within factor (feeding status - fed versus fasted, Supplementary Tables 3 and 4) using the *lme* function from the *nlme* package (R). We compared a set of models with individual predictors and combination of interacting predictors by the likelihood ratio test using *anova* function. This analysis indicates strong main effect of diet, genotype and feeding status on the blood glucose level (Supplementary Table 4, Models 2, 3 and 4) and significant genotype:diet and feeding status:diet interactions (Supplementary Table 4, Models 5 and 7 and Supplementary Fig. 4c,d). Pairwise comparisons of glucose levels in fed and fasted WT and cKO mice were performed using the generalized linear hypothesis testing (*glht*) with Tukey's contrast and Holm's correction for multiple testing (R, *muticomp* package, Supplementary Table 5).

The effect of genotype on glucose level over time in the GTT has been evaluated using mixed linear modelling with glucose as a response variable. Significant effect of genotype (*χ*^2^(1)=5.77, *P*=0.016) and time (*χ*^2^(6)=195.29, *P*<0.0001), as well as significant genotype:time interactions (*χ*^2^(6)=16.94, *P*<0.0095) have been observed. The glucose level in the cKO animals was significantly higher than in the WT at 60 min (*t*(110)=2.005, *P*=0.047), 90 min (*t*(110)=2.55, *P*=0.012) and 120 min (*t*(110)=2.55, *P*=0.021) post-injection.

### Cell culture and reagents

293T and J774A.1 cells were purchased from ATCC and cultured in Dulbecco's Modified Eagle Medium supplemented with 10% foetal bovine serum (FBS). No additional testing was performed on commercially acquired lines. BMDM were prepared from 8 to 12-week-old male mice using standard protocols. Dex, polyIC, glucose and insulin were purchased from Sigma and IL4 from R&D Systems.

### Mice

C57BL/6 mice (NCI, Charles River Laboratories) and their transgenic derivatives below were maintained in the Hospital for Special Surgery Animal Facility in full compliance with institutional guidelines approved by the HSS Animal Care and Use Committee.

The Mx1-Cre^+/+^; GRIP1^fl/fl^ (GRIP1 cKO) and matching Mx1-Cre^−/−^; GRIP1^fl/fl^ (WT) strains were generated previously^23^ by first crossing Mx1-Cre^+/+^; GRIP1^wt/wt^ (Jackson labs) to Mx1-Cre^−/−^; GRIP1^fl/fl^ mice in which GRIP1 gene Ex11 is flanked by loxP sites^42^ followed by self-crossing of heterozygotes. This GRIP1 cKO strain has been previously used to establish the role of GRIP1 as GR corepressor in the context of GR:AP1 and GR:NFκB transrepression complexes^23^.

LysM-Cre; GRIP1 mice were generated to specifically delete GRIP1 in the myeloid lineage^67^ by crossing the LysM-Cre^−/−^; GRIP1^fl/fl^ with LysM-Cre^+/+^; GRIP1^wt/wt^ to obtain double-heterozygous LysM-Cre^+/-^; GRIP1^fl/wt^ animals (LysM-Cre-Het mice; Supplementary Fig. 1a, left). Homozygous WT (LysM-Cre^−/−^; GRIP1^fl/fl^) and GRIP1 cKO (LysM-Cre^+/+^; GRIP1^fl/fl^ were produced by self-crossing LysM-Cre-Het mice. The genotype of the progeny was determined by PCR (Supplementary Fig. 3a, right) with primers listed in Supplementary Table 6.

The LysM-Cre^−/−^; GRIP1^fl/fl^ and LysM-Cre^+/+^; GRIP1^fl/fl^ mice will be made available from the corresponding author on request.

### RNA isolation and RT-qPCR

Total RNA isolation from BMDM (with QIAgen RNAeasy), random primed cDNA synthesis, qPCR with Maxima Sybr Green/ROX/ 2x master mix (Fermentas) on StepOne Plus real-time PCR system (ABI) were performed using standard protocols, and data analysed using δδCt method.

Total RNA from frozen eWAT or liver was extracted using TRIzol LS (Life Technologies). cDNA was synthesized as described above and diluted 1:20 in nuclease-free water. Samples from a cDNA pool (calibrator) were serially diluted 1:10 to 1:10^5^ to create a standard curve and relative gene expression levels were calculated based on the relative standard curve method (Guide to Performing Relative Quantitation of Gene Expression Using Real-Time Quantitative PCR, Applied Biosystems). Expression data for each gene of interest were normalized to that of *Hprt*. qPCR primers are listed in Supplementary Table 6.

### Macrophage transcriptome analysis

Total RNA from BMDM (M(con) and M(IL4); 3 biological replicates each) was polyA-enriched and converted into Illumina-compatible sequencing library with TrueSeq mRNA-Seq sample preparation kit (Illumina). Sequencing libraries from CD45+F4/80+CD11b+CD11c+CD206- ATMΦ from obese WT and cKO mice were prepared using total RNA with the SMART-Seq v3 Ultra Low Input RNA Kit (Clontech) followed by Nextera library prep protocol. Quality control of RNA and libraries was performed using BioAnalyser 2100 (Agilent). Pair-end sequencing was performed at the Weill Cornell Epigenomics Core using HiSeq2500 at the depth of ∼40 million fragments per sample. Read quality was evaluated using FASTQC. 50-bp paired reads were mapped to annotated mouse genome (mm10, build 38.75, 41,128 genes and 87,108 transcripts) with CLC Bio Genomic Workbench 7.5 software (Qiagen). Duplicated reads with more than five copies were discarded. For BMDM, differential expression was analysed using CLC implementation of EDGE algorithm^68^ with the unique exon read count as a proxy of gene expression levels. Genes with a Benjamini-Hochberg FDR-corrected *P* value<0.01 and log2 (unique exon read)>1 were considered to be differentially expressed, and shown as Reads Per Kilobase of transcript per million mapped reads (RPKM). For ATMΦ, RPKMs were used as a measure of expression. Following filtering, all genes with RPKM>1 in at least one genotype (10,126 feature IDs) were sorted by fold change relative to the expression level in the WT CD11c+ macrophages. The differences between WT and cKO were analysed by GSEA (http://software.broadinstitute.org/gsea/index.jsp) with the log ratio of the WT and cKO expression values as a ranking metric. After collapsing feature IDs into gene symbols, 7,260 genes out of 10,126 feature IDs remained for analysis. After filtering out small and large gene sets (min=15, max=500), 734 gene sets from MSigDB database v5.1 hallmark and the reactome gene sets were used for the enrichment analysis. 1,000 gene set permutations were performed, gene sets with FDR<0.1 were considered significantly enriched (see Suplementary Data for the top enriched gene sets).

### Transfections

293T cells were cultured in Dulbecco's Modified Eagle Medium+10% FBS and transfected using Lipofectamine LTX with Plus Reagent (Life Technologies). pCDNA3-Grip1 and pCS2-Klf4 expression vectors have been previously described^69^,^70^.

### Immunoblotting, immunoprecipitations and ChIP

WCE preparation from cultured cells and immunoblotting for GRIP1 (Abcam, ab10491, 1:3,000 and BD BioScience, 611319, 1:500), KLF4 (R&D System, AF3158, 1:500), PPARγ (Cell Signaling, #2443, 1:500), ARG1 (Cell signaling, #9819, 1:1,000), Vinculin (Cell Signaling, #4650, 1:1,000), FLAG (Sigma, F1804, 1:1,000), STAT6 (Santa Cruz Biotechnology, sc-981, 1:2,000) and pSTAT6-Tyr641 (Cell Signaling, #9361, 1:1,000) were performed using standard protocols. eWAT, liver and muscle tissues were homogenized with a Polytron for 20 s in lysis buffer (eWAT: 30 mM HEPES, 150 mM NaCl, 5 mM EGTA, 10% glycerol, 1% Triton X-100 and 0.5% sodium deoxycholate, pH 7.4; muscle: PBS with 1% Triton X-100) in presence of protease and phosphatase inhibitors. Phospho-Akt (Thr308; #4056, 1:1,000) and Akt (#4691, 1:2,000) antibodies were purchased from Cell Signaling. Immunoblots were quantified using ImageJ software (http://imagej.nih.gov/ij/) and normalized as described in legends.

Immunoprecipitations were performed using antibodies to FLAG (Sigma, F7425), GRIP1 (Bethyl, S300-025) or normal rabbit IgG (Santa Cruz Biotechnology) in presence of 50 μl of Protein A/G agarose beads (Santa Cruz Biotechnology). After overnight incubation at 4 °C, beads were washed five times with ice-cold lysis buffer and boiled in 50 μl of 1 × Laemmli sample buffer. Figure 2a,b show a representative of two independent experiments each.

For ChIP, cells were dual cross-linked in 2 mM disuccinimidyl glutarate solution followed by 1% methanol-free formaldehyde for 40 min total at room temperature and quenched in 0.125 M glycine for 10 min. Cells were then washed with PBS, scraped and lysed for 10 min at 4 °C in lysis buffer (50 mM Hepes-KOH pH 7.5, 140 mM NaCl, 1 mM EDTA, 10% glycerol, 0.5% NP-40 and 0.25% Triton X-100) with protease inhibitor cocktail (Sigma) and the crude nuclear extract was collected by centrifugation at 600 *g* for 5 min at 4 °C. Nuclei were washed for 10 min at 4 °C in wash buffer (10 mM Tris-HCl pH 8.0, 200 mM NaCl, 1mM EDTA pH 8.0, 0.5 mM EGTA pH 8.0 with protease inhibitors) and collected as above. Nuclei were lysed (0.1% SDS, 10 mM EDTA, 50 mM Tris, pH 8.1 with protease inhibitors) and sonicated to fragment chromatin using 17 cycles (30 s ON at high power) in a Bioruptor at 4 °C. Lysates were cleared by centrifugation (14,000 r.p.m. for 20 min at 4 °C) and incubated with antibodies to KLF4 (Santa Cruz Biotechnology, sc-20691x) or GRIP1 (Bethyl, S300-025) with 40 μl of 50% protein A/G plus-agarose per reaction at 4 °C overnight. Beads were washed four times with RIPA buffer (10 mM Tris-HCl at pH 8.0, 1 mM EDTA, 150 mM NaCl, 5% glycerol, 1% Triton X-100, 0.1% sodium deoxycholate, 0.1% SDS) and once with TE (10 mM Tris pH 8.0 and 1 mM EDTA). Each reaction was then incubated in TE-0.5% SDS-200 μg ml^−1^ proteinase K for 2 h at 55 °C, followed by 6 h at 65 °C to reverse cross-links. DNA was purified by phenol-chloroform extraction and ethanol-precipitated.

KLF4 and GRIP1 recruitment to KLF binding sites of the *Klf4* and *Arg1* genes or the GRE of *Fkbp5* was assessed by qPCR and expressed relative to signals in untreated BMDM [M(con)=1]. Primers are listed in Supplementary Table 6.

### Recombinant protein production and *in vitro* binding assays

*Klf4* coding sequence was PCR-amplified from pCS2-Klf4 expression vector with primers introducing BglII and NotI sites (Y37 5′-cgAGATCTcATGAGGCAGCCACCTGGCGAGTCTGA-3′ and Y38 5′-aCGGCGGCCGCCtAAAAGTGCCTCTTCATGTGAAGG-3′) and subcloned into pET30a His-tag bacterial expression vector (EMD Biosciences). pCDNA3-Grip1, pCDNA3-Grip1 N1007 and pCDNA3-Grip1 N765 and pCDNA3-Src1 have been previously described^69^. To create pCDNA3-HA-Grip1 765C, a XhoI fragment encoding GRIP1 765C was excised from pCDNA3-Grip1 and re-cloned into pCDNA3-HA. His-tagged KLF4 was produced in MagicMedia *E.coli* expression medium (Life Technologies) according to the manufacturer's recommendation and purified using Talon metal-affinity resin (Clontech Laboratories) as previously described^71^. GRIP1 derivatives and SRC1 were produced using the coupled *in vitro* transcription/translation system (Promega) in the presence of ^35^S-methionine, and binding assays performed as described^71^. Figure 2d shows a representative of three independent experiments.

### FA uptake assay

Macrophages were pre-incubated in FBS-free media for 90 min and the FA uptake assay was performed according to the manufacturer guidelines (Abcam, ab176768) as described^14^. Data from wells containing assay mix without cells were used as background.

### HFD model of metabolic disease

Eight-week-old male mice were placed on HFD (BioServ, F6379) or regular chow (LabDiet, 5001) for 20 weeks and weighted weekly. Following 20 weeks, animals were killed, livers and eWAT dissected and weighted.

### Histology and Image analysis

H&E staining, immunohistochemistry for F4/80 (Abcam, ab6640) and Oil Red-O staining were performed by the Laboratory of Comparative Pathology at the Weill Cornell Medical College.

Quantitative image analysis of immunostained liver slides was performed using colour deconvolution. ImageJ Colour Deconvolution 1.5 plugin^72^ performs orthonormal transformation of an acquired 24-bit RGB images using 3 × 1 vectors of individual stains' optical densities in RGB color space. This approach separates the DAB (F4/80) and haematoxylin components into individual ‘channels' that contain deconvolved information about the contribution of each stain to the total image. Each 8-bit DAB image was converted to black and white image by thresholding to capture shape/size information. The same upper threshold value (200), set to exclude background staining, was used for all measurements made on the liver slides. F4/80-stained sections of WT and cKO livers were deconvolved, and the DAB ‘channel' was quantified. The threshold intensity level was determined in comparison with a stained blank sample and was set at threefold over the background level. The area of thresholder region of interest, corresponding to DAB-stained macrophages, was determined by the Particle Analyze module of ImageJ after excluding edges and including ‘holes'. The average size of DAB-stained area corresponding to infiltrating macrophages and the ratio of the DAB-stained area to total area of the image was determined and compared between the WT and cKO by the Mann–Whitney test.

Oil Red-O-stained images were saved at 24-bit RGB and analysed using ImageJ software in the red channel containing the intensity information corresponding to Oil Red-O as described above. To compare the distributions of droplet areas between WT and cKO, we constructed area-frequency univariate kernel density estimate (KDE), a smooth function that approximates data probability density function^73^. To correct for extreme asymmetry droplet areas were log transformed; small droplets (<10 pixels) that likely correspond to debris were filtered out and the pairwise comparison of oil droplet areas distributions was performed using KDE with *sm.density.compare* function of the *sm* R package. The smoothing parameter *h* (bandwidth) has been calculated as the mean of normal optimal values^74^ for the analysed samples. To estimate the difference between KDEs, a joined density distribution was created as an average between the WT and cKO KDEs. 95% confidence band for the joined distribution representing the hypothesis of equality was constructed and permutation test with 10,000 repetitions was used to obtain the *P* value for the differences between individual KDEs and joined distribution.

### ATMΦ purification and FACS

eWAT was isolated from mice immediately after CO_2_ asphyxiation. Tissues were minced into fine (<10 mg) pieces, placed in Hanks' balanced salt solution (Life Technologies) and centrifuged at 1,000 *g* for 10 min at room temperature to pellet blood cells. An LPS-depleted Liberase 3 (Roche Applied Science) was added to the suspension (final concentration 0.04 mg ml^−1^) and the samples were incubated for 30 min at 37 °C on an orbital shaker. Digested samples were passed through a sterile 100 μm nylon mesh. The suspension was centrifuged at 1,000 *g* for 10 min and pelleted cells were collected as the SVF and resuspended in FACS buffer (1 mM EDTA in PBS-1% FBS). Macrophage staining was performed for 30 min at 4 °C using the following antibodies: F4/80, CD206, CD11c, CD11b and 7AAD (Biolegend 123114, 141710, 117309, 101207 and 420403, respectively) and CD45 (BD Pharmigen, 557659). Cells were sorted at the HSS flow cytometry core facility using FACS vantage SE (BD).

To determine the frequency of ATMΦ, the percentage of CD45+F4/80+CD11b+ cells was calculated on gated live cells. Next, the frequencies of sorted CD11c+CD206- or CD11c-CD206+ populations was calculated in the total CD45+F4/80+CD11b+ ATMΦ.

### Glucose, FFA, triglyceride, corticosterone and insulin measurements

Glucose levels were determined in whole blood from the tail vein using an automatic glucose monitor (TRUE2go Glucose Test Meter). For GTT, blood glucose levels were determined in 5 μl of blood from the tail vein in mice fasted overnight and monitored for 240 min following a glucose injection (1.5 g kg^−1^ body weight, IP). Mixed linear modelling was used to analyse GTT profiles. Areas under the GTT curves were calculated using trapezoid method using R (*trapz* with the default parameters) and compared using Mann–Whitney test. For insulin tolerance test, mice were fasted for 4 h and injected with insulin (0.75 U kg^−1^ body weight, IP). Blood glucose was measured as above right before the insulin injection (time 0) and over 120 min post-injection. Serum FFA and triglycerides were measured using quantification assay kits (Abcam, ab65341; ab65336) using mouse standards according to the manufacturer guidelines. Serum corticosterone and insulin levels were measured by ELISA (Abcam, ab108821; Crystal Chem Inc, 90080) using mouse standards according to the manufacturer guidelines.

### Data availability

RNAseq data that are described in this study have been deposited to Gene Expression Omnibus (GSE80160).

## Additional information

**How to cite this article**: Coppo, M. *et al*. The transcriptional coregulator GRIP1 controls macrophage polarization and metabolic homeostasis. *Nat. Commun.* 7:12254 doi: 10.1038/ncomms12254 (2016).

## Supplementary Material

Supplementary Information Supplementary Figures 1-6 and Supplementary Tables 1-6

Supplementary Data 1 Gene-set enrichment analysis (GSEA) data.The differences between WT and cKO were analyzed by the GSEA with the log ratio of the WT and cKO expression values as a ranking metric as described in Methods. Shown are 50 top enriched gene sets (worksheet 1) and the lists of genes corresponding to the top 6 sets plotted in Fig. 3j (worksheets 2-7).
