# Supplementary material for: The transcriptional coregulator GRIP1 controls macrophage polarization and metabolic homeostasis
Source: Nat Commun. 2016 Jul 28;7:12254. doi: 10.1038/ncomms12254 (PMC4974480; doi:10.1038/ncomms12254)
Supplement: Supplementary Information — Supplementary Figures 1-6 and Supplementary Tables 1-6 [file ncomms12254-s1.pdf]

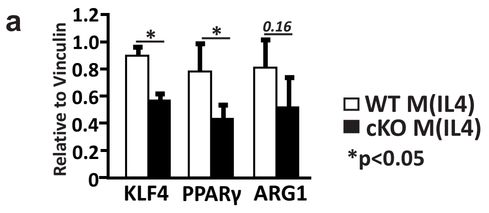

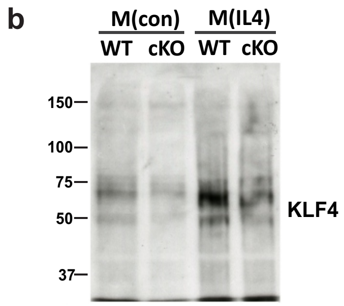

Supplementary Figure 1 | M(IL4) Protein expression quantification

(**a**) Immunoblots from Fig. 1d were scanned and quantified using ImageJ software. Signals for indicated proteins in WT and cKO M(IL4) were normalized to those of vinculin (loading control) and compared using Student t-test. Shown are average of 2-3 blots; error bars are SD.

(**b**) A full size blot of the KLF4 protein from Fig. 1d. WT and cKO M(con) and M(IL4) WCE were analyzed for the expression of KLF4 by immunoblotting.

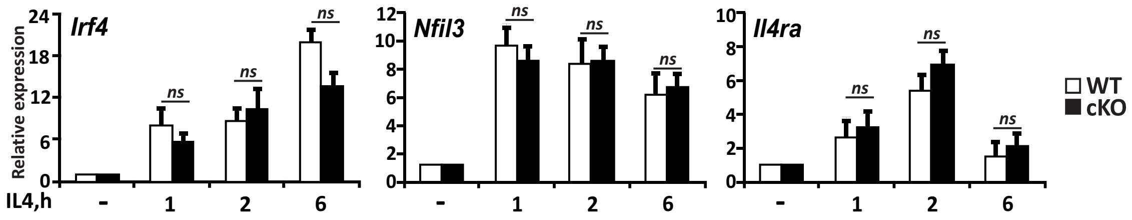

Supplementary Figure 2 | IL4-mediated STAT6 activation is intact in GRIP1 cKO BMDM
STAT6 target gene expression in WT and cKO BMDM incubated with IL4 for indicated times was assessed by RT-qPCR as in Fig. 1a. n=3 in each group; error bars are SD.

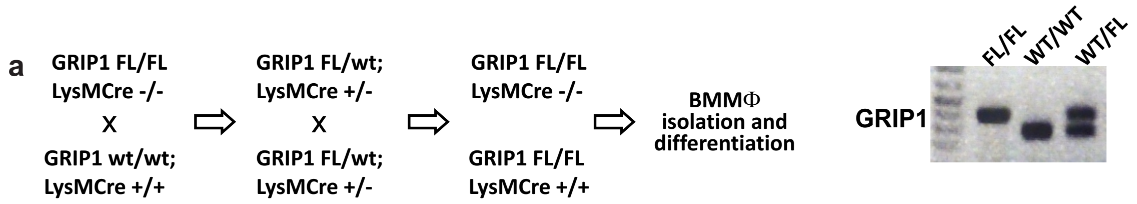

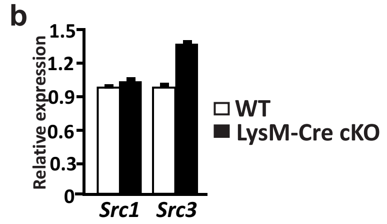

Supplementary Figure 3 | Generation of LysM-Cre GRIP1 cKO mice

(**a**) LysM-Cre$^{+/+}$; GRIP1$^{WT/WT}$ and LysM-Cre$^{-/-}$; GRIP1$^{fl/fl}$ mice were crossed as described in Methods and the progeny was PCR-genotyped at 2 wks.

(**b**) The expression of the p160 family members *Src1* and *Src3* in LysM-Cre GRIP1 cKO. Gene expression in WT and GRIP1 LysM-Cre cKO BMDM was assessed by RT-qPCR as in Fig. 1a. n>3 in each group; error bars are SD.

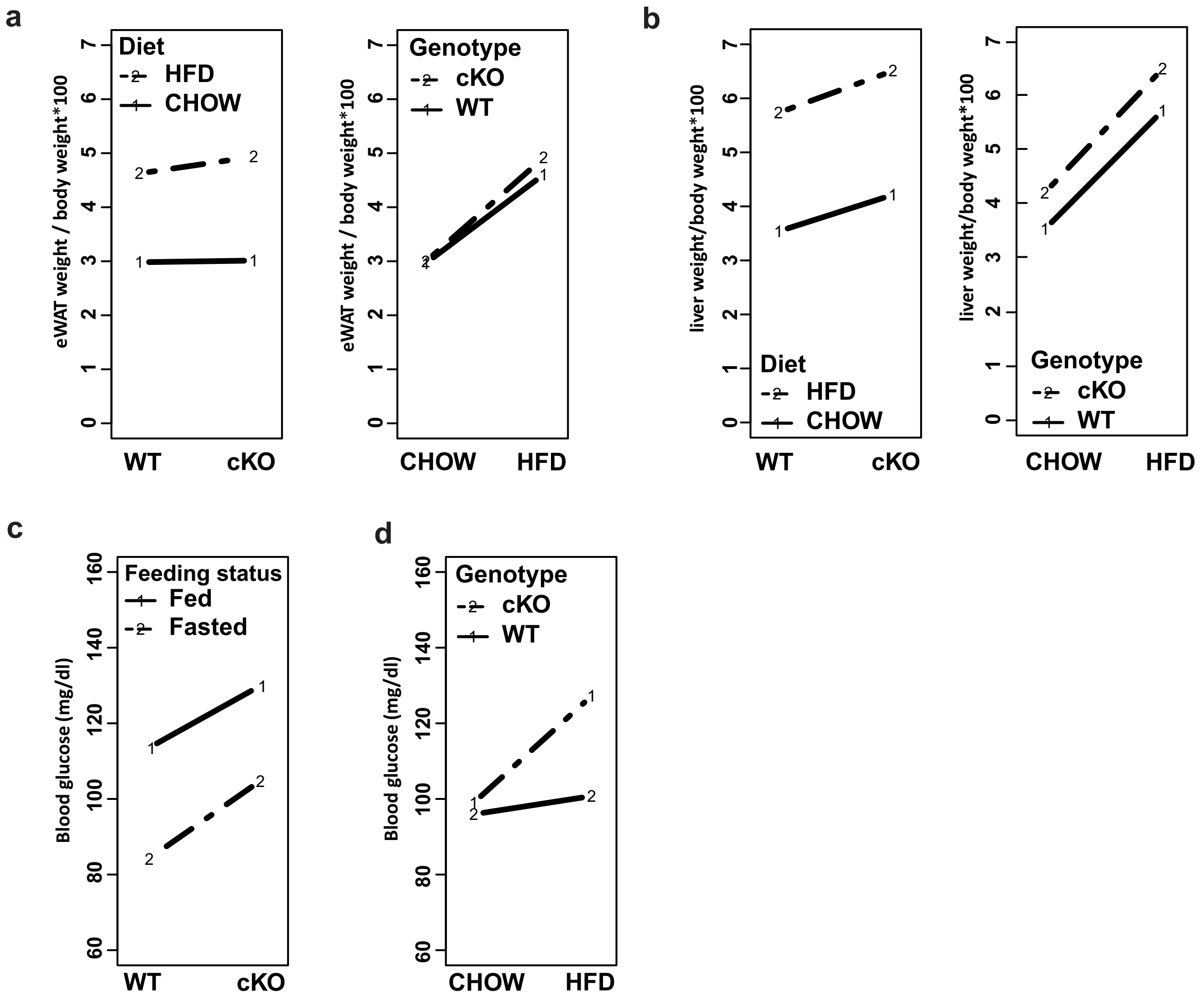

Supplementary Figure 4 | ANOVA interaction plots for eWAT weights, liver weights and blood glucose levels
Uncorrected means for the respective rows and columns (see Methods) were used to build interaction plots.
(**a**) In a 2x2 ANOVA design, no interactions between genotype:diet and significant diet effect has been observed for both WT and cKO with regard to the normalized eWAT weight (see Methods and Supplementary Table 1).
(**b**) In a 2x2 ANOVA design, no interactions between genotype:diet and significant diet effect has been observed for both WT and cKO with regard to the normalized liver weight (see Methods and Supplementary Table2).
(**c**) No interactions between genotype and the feeding status (fed *vs.* fasted) and a significant main effect of feeding status on the blood glucose level (see Methods and Supplementary Table 3, 4).
(**d**) Interactions between genotype and diet (chow *vs*. HFD) show significant main effect of genotype on the blood glucose level (see Methods and Supplementary Table 3, 4).

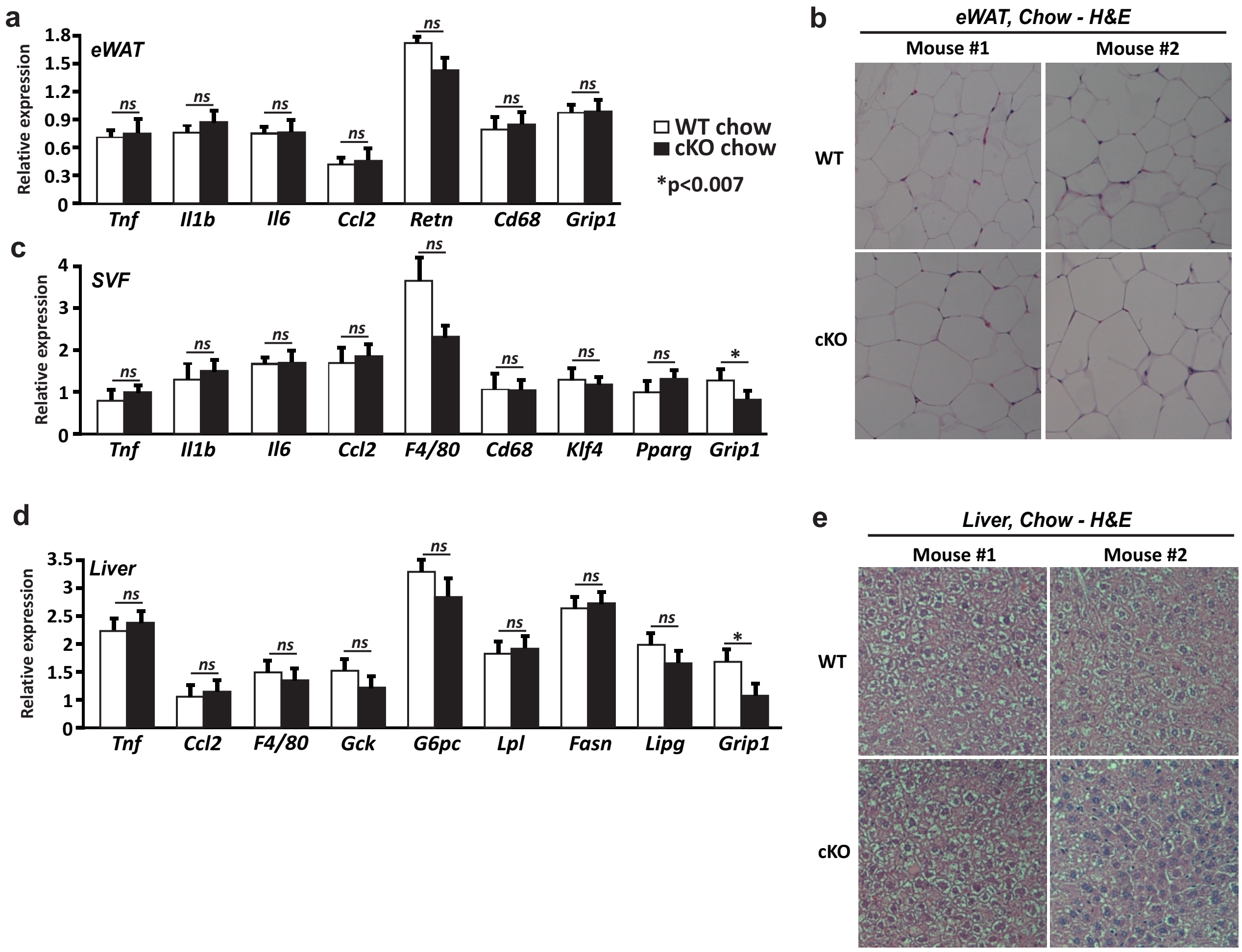

Supplementary Figure 5 | Chow-fed GRIP1 cKO mice display no apparent phenotype in metabolic tissues

(**a**) Total RNA was isolated from the eWAT of WT and GRIP1 cKO mice (n>5 in each group) and the expression of indicated genes was assessed by RT-qPCR as in Fig. 3e.

(**b**) Representative H&E staining of WT and GRIP1 cKO eWAT of chow-fed mice was performed as in Fig. 3d.

(**c**) Total RNA was isolated from the SVF of WT and GRIP1 cKO chow-fed mice (n=7 in each group) and the expression of indicated genes was assessed by RT-qPCR as described in Fig. 3f.

(**d**) Total RNA was isolated from the livers of WT and GRIP1 cKO mice (n>5 in each group) and the expression of indicated genes was assessed by RT-qPCR as in Fig. 3e.

(**e**) Representative H&E staining of WT and GRIP1 cKO livers of chow-fed mice was performed as in Fig. 3d.

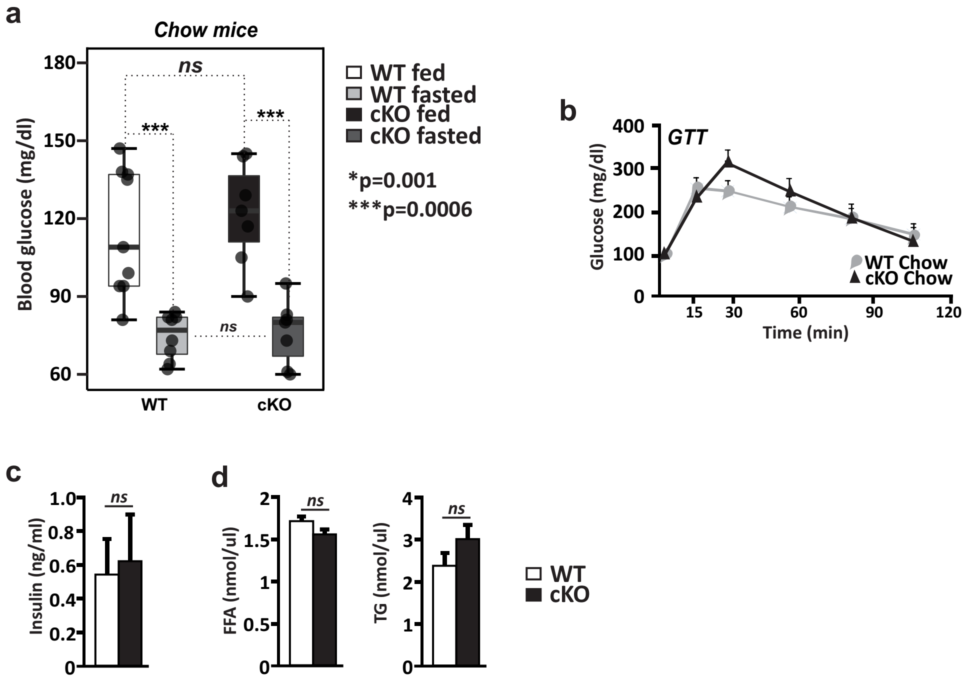

Supplementary Figure 6 | Chow-fed GRIP1 cKO mice have normal glucose tolerance

(**a**) Blood glucose levels in WT and GRIP1 cKO mice during *ad libitum* chow feeding or upon overnight starvation. n>6 in each group. The differences between fed and fasted mice within and between genotypes were evaluated by mixed linear modeling as described in Methods. The statistical significance of pairwise comparison of means was evaluated using Tukey's contrast with Holm's corrections for multiple comparisons.

(**b**) GTT in chow-fed WT and GRIP1 cKO mice. GTT was performed as in Fig. 5b. n=5 in each group.

(**c**) Insulin levels were measured by ELISA in WT and cKO serum after 20 wks of chow diet. n=6 in each group; error bars are SD.

(**d**) Serum levels of FFA and TG in chow-fed WT (n=7) and GRIP1 cKO (n=6) mice were compared using Student t-test. Error bars are SEM.

Supplementary Table 1.

ANOVA table for the effect of diet type and genotype on the normalized weight of eWAT

	Df	Sum Sq	Mean Sq	F value	Pr(>F)
genotype	1	0.088	0.088	0.144	0.708
diet	1	21.297	21.297	**34.709**	**4.45e-06**
genotype:diet	1	0.132	0.132	0.216	0.647
Residuals	24	14.726	0.614		

Supplementary Table 2.

ANOVA table for the effect of diet type and genotype on the normalized weight of livers

	Df	Sum Sq	Mean Sq	F value	Pr(>F)
genotype	1	3.24	3.24	2.774	0.109
diet	1	33.55	33.55	**28.750**	**1.67e-05**
genotype:diet	1	0.02	0.02	0.014	0.905
Residuals	24	28.01	1.17		

Supplementary Table 3.

Summary of factorial design for the analysis of the effect of diet, genotype and feeding status on blood glucose levels

**Genotype**	**Diet**	**FED**	**FASTED**	Row means
WT	CHOW	114.889	74.625	94.757
	HFD	112.625	88.875	100.75
cKO	CHOW	121.857	76.143	99
	HFD	133.533	120	126.77
	Col.means	120.726	89.911	

Supplementary Table 4.

Mixed linear modeling of the effect of diet, genotype and feeding status on blood glucose levels

	Model	df	AIC	BIC	logLik	Test	L.Ratio	p-value
Base	1	3	862.6773	870.2099	-428.3387			
Genotype(Gen)	2	4	853.0791	863.1226	-422.5396	1 vs 2	11.59817	0.0007
Gen+feeding_status	3	5	820.2041	832.7584	-405.1021	2 vs 3	34.87502	<.0001
Gen+feeding_status+diet	4	6	812.3764	827.4415	-400.1882	3 vs 4	9.82774	0.0017
Model 4 + Gen:feeding_status	5	7	813.9193	831.4954	-399.9597	4 vs 5	0.45704	0.4990
Model 5 + Gen:diet	6	8	809.9472	830.0341	-396.9736	5 vs 6	5.97216	0.0145
Model 6 + feeding_status:diet	7	9	802.6531	825.2508	-392.3265	6 vs 7	9.29409	0.0023
Model 7 + Gen:feeding_status:diet	8	10	803.3387	828.4473	-391.6693	7 vs 8	1.31443	0.2516

Supplementary Table 5.

Pairwise comparison of the glucose levels in fed and fasted WT and cKO mice

Contrasts	p values
fasted.CHOW.KO - fed.CHOW.KO	4.45E-06
fasted.CHOW.WT - fed.CHOW.WT	1.13E-05
fed.CHOW.WT - fed.CHOW.KO	1
fasted.CHOW.WT - fasted.CHOW.KO	1
fasted.HFD.KO - fed.HFD.KO	0.38
fasted.HFD.WT - fed.HFD.WT	0.00048
fed.HFD.WT - fed.HFD.KO	0.018
fasted.HFD.WT - fasted.HFD.KO	5.24E-05

Supplementary Table 6. Primers used in this study.

Number	Expression Primers	Sequence
y81	Arg1_F	ACCACACTGACTCTTCCATTCTT
y82	Arg1_R	TTGATGTCCCTAATGACAGCTCC
n44	Ccl2_F	AGGTCCCTGTCATGCTTCTGGG
n45	Ccl2_R	CCTCATTGGGATCATCTTGCTGGTG
c66	Cd163_ F	ATGCCCAACTTCTATTAAAGCCC
c67	Cd163_ R	TTCCCCACCCATCATGTTTGC
t18	Cd36_F	ATAGGGTGGGGCAGCTATCA
t19	Cd36_R	TGCACACTTGGTTTTGCCAT
y79	Cd68_ F	CTTCCCACAGGCAGCACAG
y80	Cd68_R	AATGATGAGAGGCAGCAAGAGG
y77	F4/80_R	CTTTGGCTATGGGCTTCCAGTC
y78	F4/80_R	GCAAGGAGGACAGAGTTTATCGTG
v28	Fasn_F	GATGACATGAACATTGGAGCC
v29	Fasn_R	TGAGATCCCAGCACTTCTTG
v34	G6pc_F	GTTTCGCGCTTGGATTCTAC
v35	G6cpc_R	CAAAGGGTGTAGTGTCAAGGT
v22	Gck_F	GGAATACATCTGGTGTTTCGTC
v23	Gck_R	TCCTTAGACCTGGGAGGAAC
p30	Grip1_F	CACCTCGCTCAAGGAGAAGCATAAG
p31	Grip1_R	GCTCCTGTTTGACAGTCACTTCCG
k48	Il1b_F	GTAGCTGCCACAGCTTCTCCACAGCC
k47	Il1b_R	GGGCTGCTTCCAAACCTTTGACC
z72	Il4ra_F	CCTGCCATGTTCTGCTAGTC
z73	Il4ra_R	ATCCCAGAGGGGCACCTAAA
k74	Il6_F	CCGCTATGAAGTTCCTCTCTGCAAG
k75	Il6_R	GCCTCCGACTTGTGAAGTGGTATAGAC
y4	Irf4_F	GCCTGTATTACCGGGACATC
y5	Irf4_R	GGTCCAGGTTGCTAACATCA
o89	Klf4_F	CCTTGGTGTTAGCTGAGATTG
o90	Klf4_R	GAACAGGGAGGGAGAGAGAAA
v12	Lipg_F	ACCGAAGGCAAACCTGAACA
v13	Lipg_R	GGCCTCAGACTCACAAGCAT
v6	Lpl_F	AAATGCTGCCTGGGTTCAGT
v7	Lpl_R	CAACTAACCTGCCCTGTGGT
t40	Mrc1_F	CCCAAATCCTCCAACCCAGAT
t41	Mrc1_R	GATTTCCTCCTCCCCACCAT
p46	Nfil3_F	CGGTGGAGCACACTCAGGAAAGC
p47	Nfil3_R	CAAAGCTCTCCAACTCCACGGGC
y51	Vcam1_F	GCCGGAATCGTCCCTTTTTG
y52	Vcam1_R	TGTTGCTTTCCAGTTGCCCC
t50	Pparg_F	ACCATTGAATAAATGCTCCTGAGA
t51	Pparg_R	CTTGGCCTCCCACAGACATC
y14	Retn_F	CCTCACCCTGTTCCAACCCA
y15	Retn_R	TTTGGAGTGAGGGGAGGTTTG
r82	Src1_F	TGGAGCAAGAGAAATCCACG
r83	Src1_R	TCCAAAGCCTCCAGAAGAAG
r84	Src3_F	TTCCTGAGCAACACAGATGC
r85	Src3_R	ATTACTGCTGCTTCTTGGCC
e1	Tnf_F	TCTAAGAGCTCTGTCTTTTCTCAGCC
d99	Tnf_R	CCAGGATCATCTTCTCAAAATTCGAGTG

Number	ChIP Primers	Sequence
x35	Arg1 ChIP_F	TCACGCGTGGTAGCCGACGAGAG
x36	Arg1 ChIP_R	CGCACGCGTAAAGTGGCACAACTCACGTA
w17	Klf4 ChIP_F	ACGTGCGCGGAGTTTGTTTATTTAGC
w18	Klf4 ChIP_R	CTTATAACTTCTCGCTCGCTTGCTC
p95	Fkbp5 ChIP_F	GCACATCAAGTGAGTCTGGTCACTGC
p96	Fkbp5 ChIP_R	TGCCAGCCACATTCAGAACAGGG

Number	Genotyping Primers	Sequence
l45	Grip1 genotype control_ F	CTAGGCCACAGAATTGAAAGATCT
l46	Grip1 genotype control_ R	GTAGGTGGAAATTCTAGCATCATCC
l47	MX1 Cre_F	GCGGTCTGGCAGTAAAAACTATC
l48	MX1 Cer_R	GTGAAACAGCATTGCTGTCACTT
x63	LysM Cre_F	CTTGGGCTGCCAGAATTTCTC
x64	LysM Cre_R	CCCAGAAATGCCAGATTACG
y85	LysM Cre_R	TTACAGTCGGCCAGGCTGAC
